# Optical maneuvering of dandelion-inspired fliers with vortex-enabled stability

**DOI:** 10.1126/sciadv.aee8014

**Published:** 2026-07-29

**Authors:** Jianfeng Yang, Soumarup Bhattacharyya, Aditya Potnis, Ahmet Gungor, Hao Zeng, Ignazio Maria Viola

**Affiliations:** ^1^ Faculty of Engineering and Natural Sciences, Tampere University, P.O. Box 541, FI-33101 Tampere, Finland.; ^2^ School of Engineering, Institute for Energy Systems, University of Edinburgh, Edinburgh EH9 3FB, UK.; ^3^ Department of Industrial Engineering, Alma Mater Studiorum, University of Bologna, 47121 Forlì, Italy.

## Abstract

Maneuvering untethered, centimeter-scale airborne structures has been a long-standing challenge. Active flight systems, relying on high-power-density actuators alongside mechanical and electronic components, are constrained by critical limitations in energy delivery and miniaturization. In contrast, passive systems transported and distributed by the wind typically lack the capability for mid-air controlled maneuverability. Here, we report an ultralight (1.2 milligrams) hexagonal polymeric assembly capable of passive flight with optical control of its trajectory. This dandelion-inspired drone, dandidrone hereafter, incorporates six radially arranged filamentous structures, of which morphology is dynamically controlled through photomechanical deformation by six independent soft actuators made of liquid crystalline elastomer thin films. Compared to the diaspore of the dandelion, dandidrones demonstrate a similar terminal velocity (∼0.5 meters per second), 45% better positional stability and nearly zero rotational rate (1.68 ± 1.0° per second; natural seeds: 50.8 ± 17.7° per second). Particle image velocimetry and computational fluid dynamics simulation reveal that a stable asymmetric separated vortex ring underlies its flight stability, enabling mid-air steerability. When free-falling in a low-turbulent airstream, the light-driven hexapodal fliers demonstrate precise altitude control, reversible body flipping, pattern formation, interactive swarm, and controlled trajectories across the three-dimensional space. The results show that responsive materials with light-induced asymmetry can bring about maneuverability in air, paving the way for agile, untethered controlled microfliers.

## Teaser

Light steers a dandelion-inspired drone through stable, controlled 3D flight enabled by vortex dynamics.

## INTRODUCTION

Micro- and nanodrones have undergone a transformative evolution, progressing from rudimentary motor-driven prototypes (*1*) to advanced piezoelectric (*2*) and dielectric-actuated systems (*3*) and, ultimately, to sophisticated platforms using novel actuation paradigms (*4*–*7*). This developmental trajectory has been driven by interdisciplinary advancements spanning engineering innovations, insights from biological flight strategies (*8*, *9*) and materials sciences (*6*, *10*, *11*). Among these, active drones that rely on onboard actuators or propulsion units for thrust and maneuverability represent a pinnacle of engineering miniaturization (*12*, *13*). However, they are limited by the need for high power density and the complexities of mechanical transduction (*5*). To meet their power demands, these systems often resort to tethered setups that undermine steerability and operational range (*4*, *5*, *8*). Moreover, achieving compact integration of power units, control electronics, and actuation elements limits the development of agile, untethered drones with autonomous flight capabilities (*14*, *15*).

In contrast, nature offers an energy-efficient alternative for centimeter-scale flight: Wind-dispersed seeds are passively transported and distributed by the wind and remain airborne without relying on active propulsion (*16*, *17*). For example, maples exploit autorotation to generate lift and decrease the terminal velocity (*18*, *19*), whereas dandelions exploit a highly porous filamentous pappus to form a separated vortex ring (SVR) (*20*) associated with enhanced drag. The maple’s lift and the dandelion’s enhanced drag allow these diaspores to fall slowly and be uplifted by turbulent updrafts, remaining afloat for hours and being transported for hundreds of kilometers (*21*, *22*). The intrinsic efficiency of these passive locomotion mechanisms circumvents many of the energy issues that limit active systems while simplifying design, reducing structural size and weight (*6*, *23*–*29*).

Notwithstanding the abovementioned advantages, achieving control in passive flight presents critical engineering challenges. The principal limitation resides in enabling untethered morphology control to attain precise maneuverability within the three-dimensional (3D) space while retaining aerodynamic stability and control of the trajectory. Rapidly deformable, light-responsive materials have been demonstrated to enable takeoff and landing actions (*26*, *30*), but efforts to extend this maneuverability to sustained aerial navigation have been hampered by the instability issue. The relatively low inertia of these devices compared to heavier powered drones often results in erratic motion patterns, including nonstop spinning and large-scale oscillations (*26*), thereby complicating the maintenance of a stable flight path.

## RESULTS

### System concept

**Fig. 1.**
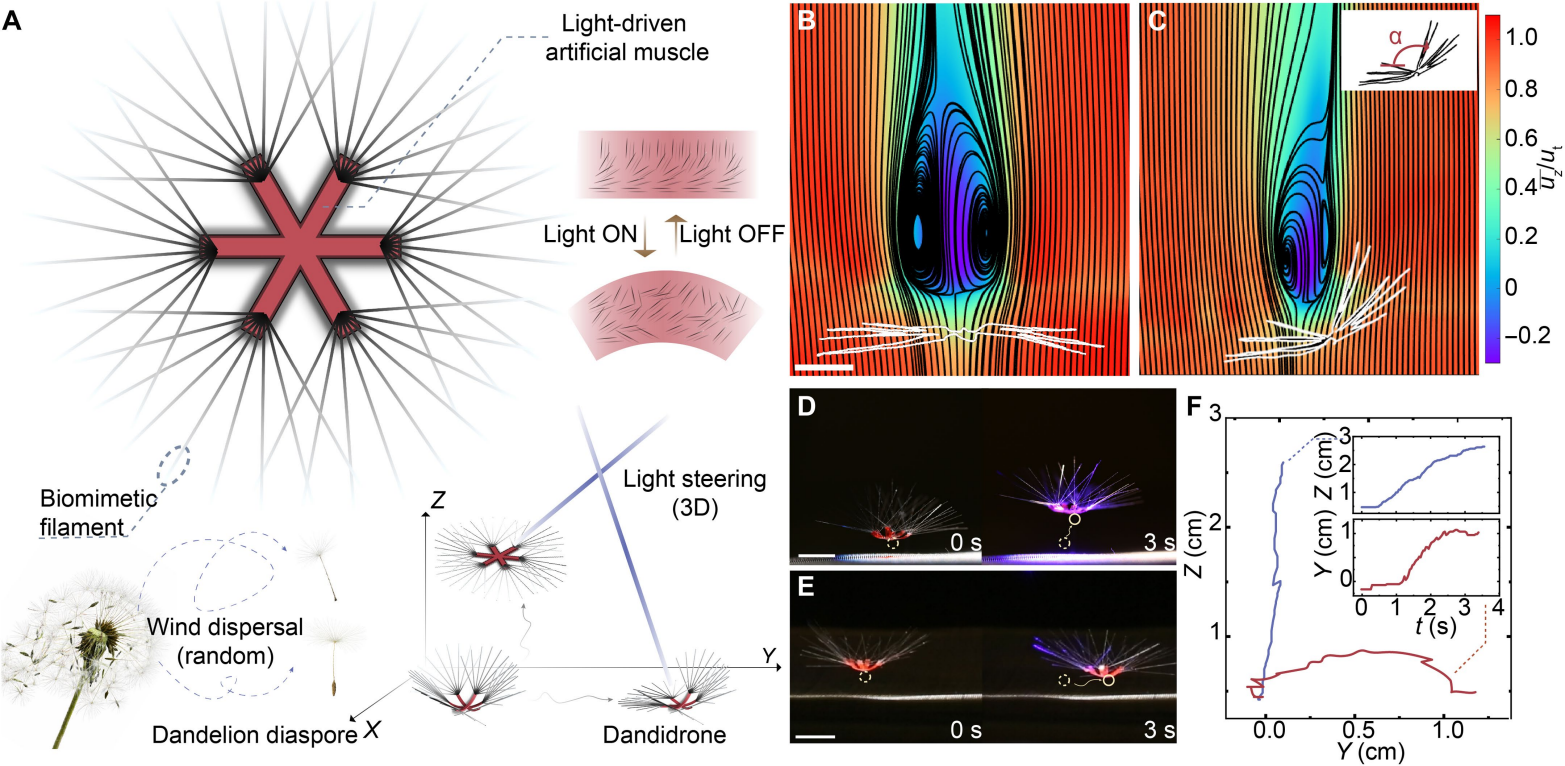
System concept. (**A**) Schematics of the dandidrone and that of its light-driven artificial muscle when the external illumination is on and off. The dandelion image in the bottom-left corner of (A) is a real photograph taken in the laboratory. Ratio of the magnitude of the time-averaged vertical flow velocity ($\bar{u_{z}}$) and that of the terminal velocity *u*_t_ on a vertical plane through the center of the dandidrone for an opening angle: (**B**) α = 180° and (**C**) α = 120° (asymmetric configuration). Light-induced (**D**) upward and (**E**) sideward displacement of the dandidrone. The wind tunnel speed in (D) and (E) is 0.6 m s^−1^. (**F**) Upward (blue) and rightward (red) trajectories corresponding to (D) and (E), respectively, with their time evolution presented in the insets. Scale bars, 0.5 cm. The light intensity is 700 mW cm^−2^.

Here, we report a dandidrone with unprecedented aerodynamic stability and steering capability. Figure 1A shows the hexapodal flier inspired by the radially distributed porosity of the pappus of the dandelion’s diaspore. It features six centro-aligned robotic arms made of artificial muscles. Each arm is uniformly tipped with nine biomimetic filaments made of fabric fiber. To achieve untethered light actuation, a liquid crystal elastomer (LCE) (*31*, *32*) strip is used as an artificial muscle to induce mechanical deformation. The details of design, photothermal actuation mechanism, and optical properties of LCE are reported in note S1 (figs. S1 to S17). The six centro-aligned LCE arms design was optimized through a systematic increase in mid-air stability and actuation efficiency. Two/four-arm designs enabled basic takeoff control but exhibiting instability in mid-air (fig. S3). Elevating the number of actuator arm enhances the symmetry, improving the stability (fig. S4); however, the reduced spacing between adjacent arms poses hurdles on individual actuation. The six-arm design therefore represents an optimal solution for this study. In the present system, the light-responsive actuator exhibits a blocking force of 0.5 to 3 mN (fig. S11), a response time of ∼1 s (fig. S12), a power density 0.11 W kg^−1^, and an energy conversion efficiency on the order of ∼3 × 10^−6^ (fig. S13).

Alike the filamentous pappus of the dandelion diaspore (fig. S17), the hexapodal structure enables the generation of an SVR (Fig. 1, B and C) that enhances the aerodynamic drag and diminishes the terminal velocity, $u_{t}$ (in the closed state, $u_{t}$ = 0.73 ± 0.04 m s^−1^; in the open state, $u_{t}$ = 0.44 ± 0.02 m s^−1^; for the natural dandelion diaspore, $u_{t}$ = 0.5 ± 0.05 m s^−1^). Details of the SVR are reported in note S2 (figs. S18 to S26). The drag coefficient (*C*_D_) of dandidrones within a range of sizes (diameter *d* from 1.5 to 3 cm) and weights (from 1.12 to 4.02 mg) were evaluated through drop tests in quiescent flow (details in fig. S27 and Materials and Methods). The opening angle α between filaments at opposite ends can be controlled by varying the excitation light intensity, yielding to a reduction in $u_{t}$ by more than 2/3 when filaments are turned from closed (vertical, α = 0°) to open (horizontal, α = 180°) (fig. S28). Furthermore, the tenable initial curvature of the LCE allows it to be programmed into either a curved or flat configuration (*33*). This design flexibility enables the dandidrone to adopt two distinct initial structural states: a closed or open configuration. Light-triggered ascent or descent can be achieved through variations in *C*_D_ and $u_{t}$ by morphing the drone structure (fig. S29).

To investigate the aerodynamics of the dandidrone, a bespoke vertical wind tunnel (fig. S30) was built to produce a uniform upward airflow matching the terminal velocity, so that the dandidrone hovers at a constant height in the wind tunnel (Materials and Methods). Dandidrones with any α exhibit remarkable stability as compared to natural seed diaspore (fig. S31). The stability metric is evaluated by the SD (σ) of the horizontal (*Y*) and vertical (*Z*) position over a 1-s time interval measured at 25 Hz. Specifically, whereas the natural dandelion diaspore exhibits σ = 0.028 cm (*Z* axis; *n* = 10 independent biological repeats), the dandidrone shows σ = 0.016 cm (*Z* axis; *n* = 5 repeated measurements of the same sample), i.e., 45% less than the natural counterpart (fig. S31). Furthermore, the dandidrone has ultrahigh angular stability around its vertical axis compared to the dandelion diaspore. Whereas the spinning rate of the dandelion diaspore is 50.8 ± 17.7° s^−1^ (*n* = 10), that of the dandidrone is only 1.68 ± 1.0° s^−1^ (*n* = 5; fig. S32). The stability of airborne position and a nearly zero spin are the key prerequisites for enabling complex, multimode motion control, which will be illustrated later.

Particle image velocimetry (PIV) is used to measure the flow field around the dandidrone (Materials and Methods). For convenience, PIV measurements are taken with the dandidrone kept fixed at a constant position and with the wind tunnel velocity set at the terminal velocity observed with a free-falling dandidrone. We demonstrated that the shape and size of the SVR, which is a toroidal vortex in the wake, can be controlled through the opening angle α and by inducing asymmetry in the filamentous structure (*34*, *35*) (Fig. 1, B and C). Details of aerodynamic measurement are reported in note S3 (figs. S27 to S32). The SVR size grows with increasing opening angle, as revealed by the increasing distances between both its nodes and the saddle points on a vertical section across its center (fig. S25). Uniform illumination induces structural opening in an initially closed dandidrone (Fig. 1D and movie S1), resulting in a larger SVR and higher drag, and thus uplifting the dandidrone in the wind tunnel (displacement data in Fig. 1F). Light-induced vertical motion in both configurations is observed in detail for various light intensities (figs. S33 to S35).

Moreover, impulsively providing a light excitation on one side of the dandidrone results in the illuminated filament group opening abruptly, generating a lateral force propelling the dandidrone away from the light, as shown in Fig. 1 (E and F) and in movie S2. Details of asymmetric shape-morphing (fig. S36), lateral moving speed (figs. S37 and S39) and light intensity threshold (figs. S38 and S40) reveal the trajectories and the variation in locomotion speed of the dandidrone at different light intensities.

### Airborne stability

**Fig. 2.**
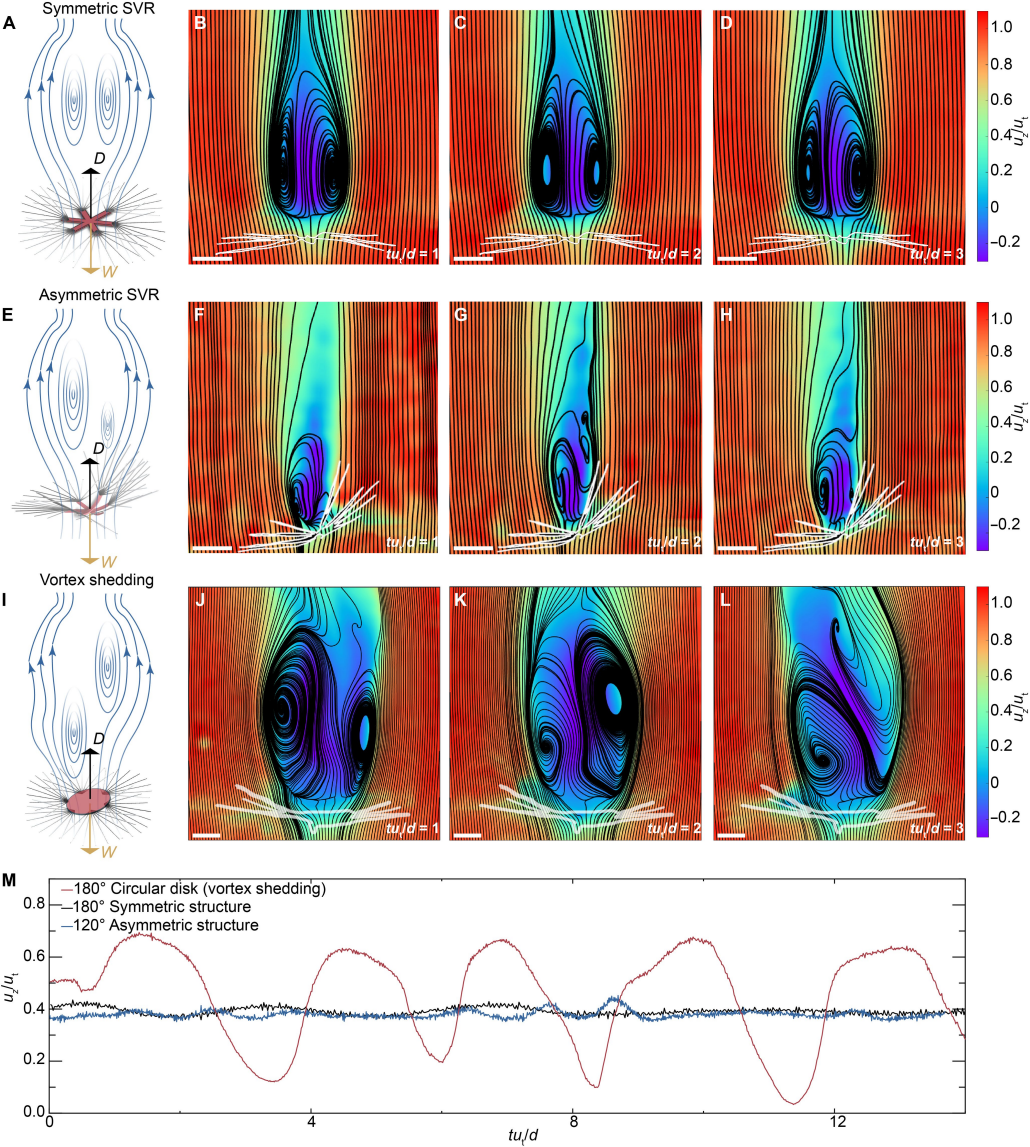
Stability of the SVR. Schematics and instantaneous flow fields of (**A** to **D**) the symmetric dandidrone configuration with an opening angle α = 180° (fully open configuration), (**E** to **H**) the asymmetric configuration with α = 120°, and (**I** to **L**) the fully open dandidrone (α = 180°) with the addition of a 1-cm-diameter disk at its center. The instantaneous flow fields show the contours of the ratio of the magnitude of the instantaneous vertical flow velocity (*u*_z_) and that of the terminal velocity and the streamlines constrained on the vertical plane through the center of the dandidrone for three instants, each at one convective period apart (*tu*_t_/*d* = 1, 2, and 3). (**M**) Time series of the velocity magnitude one diameter downstream of the center of the model for the three configurations. Scale bars, 0.5 cm.

For a dandidrone in steady fall, the weight (*W*) is balanced by the aerodynamic drag (*D*), which increases with increasing velocity (Fig. 2A). To ensure high endurance, the mean terminal velocity must be as low as possible. Furthermore, the stability of the wake, and that of the SVR in particular, ensures that the dandidrone falls steadily, as opposed to chaotically, flattering, tumbling, etc. A steady fall is a critical requirement for precise steerability. To this end, we have optimized the total number of filaments as filament density controls both porosity and effective permeability of the structure. To illustrate how filament number affects flight stability, we present computational fluid dynamics (CFD) simulations of flier wakes across varying permeabilities. In our model, the dandidrone consists of a central hexapod surrounded by a permeable disk representing the filaments. The disk’s permeability is related to its average porosity and is therefore inversely related to the filament count; this is quantified using a nondimensional Darcy number *Da*. To examine the effect of filament number, and thus permeability, on wake stability and its impact on flier dynamics, we performed simulations across a range of *Da* values. Specifically, *Da* = 10^−6^ represents an effectively impermeable disk corresponding to a flier with a very large number of filaments. The case with *Da* = 10^−1^ represents a hexapod with only a few filaments. Last, *Da* = 10^−3^ corresponds to optimal permeability and an intermediate number of filaments alike the one we adopted in the physical structure. The source of vortex shedding is a strong shear layer in the impervious disk and the hexapod with few filaments; this is evident from instantaneous streamlines showing asymmetric or chaotic wakes (fig. S18, A and C). The intense shear layer is unstable and rolls up into large-scale vortices that are shed in the wake, resulting in classic vortex shedding. This leads to nonzero, oscillating instantaneous aerodynamic pitching moments ($C_{M}^{Y}$) on the fliers (fig. S18D), which cause unstable free fall as observed experimentally. For the intermediate *Da* = 10^−3^ (fig. S18B), representing an optimal filament count, moderate permeability allows flow bleed-through that reduces velocity gradients in the hexapod-generated shear layer. This results in the less intense, and more stable shear layer, producing symmetric streamlines with a pronounced steady vortex ring. The stabilized wake yields near-zero pitching moments and balanced lateral forces on dandidrones with SVR stabilization, enabling steady descent. Isosurfaces of *Q*-criterion (fig. S19 and movie S3) further illustrate that only fliers within an optimal *Da* range, and thus optimal filament numbers exhibit steady wakes with stable SVRs. These findings are confirmed with experimental drop tests (fig. S20). Fliers with only a hexapod or very few filaments fall irregularly due to insufficient stabilization of shear layers; conversely, those with too many filaments exhibit chaotic descent due to strong vortex shedding. Only fliers within an optimal filament range (e.g., 30 to 54 filaments) demonstrate consistently steady descent. Furthermore, to investigate the stability of the SVR, contours of instantaneous vertical velocity and streamlines on a vertical plane through the dandidrone’s center are taken at different time instants (Fig. 2, B to D). The small differences in the flow fields at different times confirm that the SVR is stable, similar to that of the dandelion diaspore. The SVR stability persists regardless of variations in the opening angle for both symmetrically oriented models (fig. S24) and asymmetric configurations (*36*) (Fig. 2, E to H).

As a control, Fig. 2 (J to L) displays contours of instantaneous flow speed and streamlines for a fully open symmetric dandidrone incorporating an impervious, 1-cm-diameter disk at its center (*37*, *38*) (Fig. 2I). Unlike the consistent wake topology observed in the dandidrones, the disk’s wake is unstable, resulting in periodic vortex shedding under the same velocity conditions (movie S4). This is evident by the low amplitude of the time series of the vertical velocity measured one diameter downstream of the dandidrone with two different opening angles compared to the large amplitude oscillations of the vertical velocity in the wake of the dandidrone with the added disk (Fig. 2M). Quantitative analyses of vorticity and detailed comparison between symmetric and asymmetric dandidrones, as well as hexapodal cores without filaments and impervious disks, are given in fig. S26 and movie S4. These findings underscore the pivotal influence of the permeability and structural asymmetry on the vortex dynamics.

### 3D locomotion

**Fig. 3.**
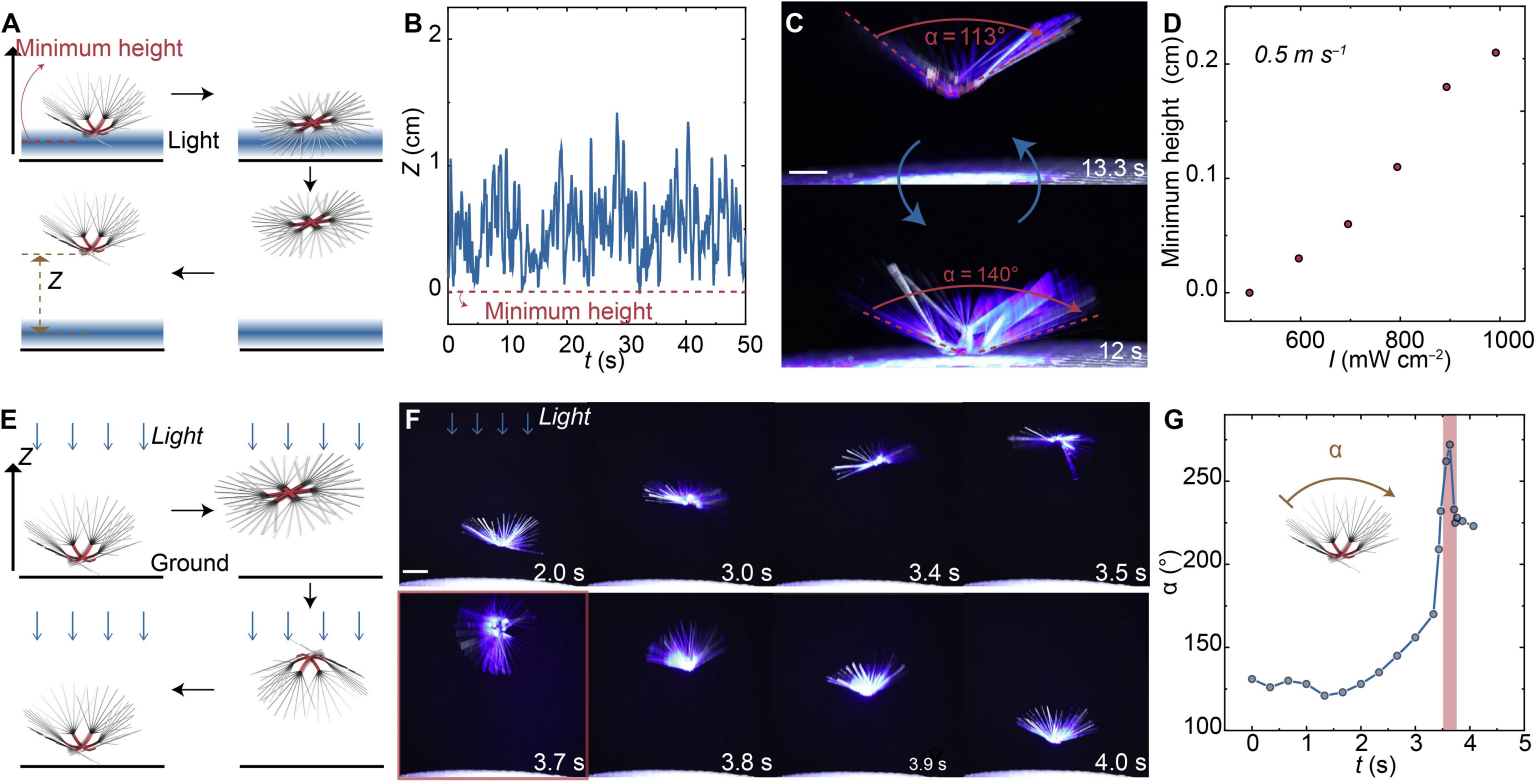
1D locomotion of the dandidrone. (**A**) Schematic drawing of the dandidrone with an optically set low-altitude limit. (**B**) Time history of the *Z* coordinate of the dandidrone from the axis of the laser beam. (**C**) Snapshot images of the dandidrone’s self-regulating opening angle while oscillating around the height limit. Terminal velocity without illumination: 0.8 m s^−1^. (**D**) Minimum height of the dandidrone for different light intensities in a wind tunnel speed of 0.5 m s^−1^. (**E**) Schematic of the light-controlled flipping motion of the dandidrone. (**F**) Snapshots of the flipping motion of the dandidrone. (**G**) Time evolution of the opening angle α during the flipping process. Wind tunnel speed: 0.6 m s^−1^. Light intensity: 650 mW cm^−2^. Scale bars, 5 mm.

Here, we demonstrate the multimodal maneuvering of dandidrones in the 3D space. First, the control of the vertical position of a symmetric dandidrone free flying in a uniform constant upward flow stream is demonstrated, facilitated by the photomechanical change in the opening angle. The altitude *Z* can be controlled by actively switching on and off the light to symmetrically increase and decrease the opening angle, respectively (Fig. 1F). However, if a constant illumination is provided below the target altitude (*Z*_0_) and obscured above that height, the dandidrone would automatically settle at the target altitude *Z*_0_. This procedure, which is demonstrated in Fig. 3 (A and B), allows positioning the dandidrone at a certain height without actively controlling or tracking its position. Consider an initially closed dandidrone in the illuminated region (*Z* < *Z*_0_). It opens because the provided illumination causes the opening angle and the drag to increase, and thus it moves upward. When it passes the target height (*Z* > *Z*_0_), it is out of the light beam, and thus the structure closes, reducing the drag and making the dandidrone to move downward toward the target position (Fig. 3C). Overall, the dandidrone moves upward when below *Z*_0_ and downward when above it, resulting in a vertically oscillatory kinematics near the desired altitude, which serves as a tenable upper or lower bound depending on the initial configuration (Fig. 3D and figs. S41 to S43).

A high excitation light intensity causes the opening angle to exceed 180°. The resulting equilibrium position is unstable because the center of gravity is above the aerodynamic center, and thus the dandidrone undergoes a spontaneous flipping to bring the center of gravity below the filaments [Fig. 3 (E and F) shows the schematic drawing and snapshots of the flipping motion, respectively; more details are shown in movie S5, and the tracking of trajectory is shown in fig. S44]. The shape-morphing induced instability can be qualitatively assessed by tracking the opening angle α (inset of Fig. 3G). The dandidrone flips as α reaches 260° at 3.5 s. Thereafter, α stabilizes at ∼220°; the light now illuminates the opposite side of the drone. (Fig. 3G). After ceasing the light, the dandidrone flips back and returns to its original configuration. The flipping is predictable and repeatable, as demonstrated by a series of 11 successive cycles in fig. S45.

**Fig. 4.**
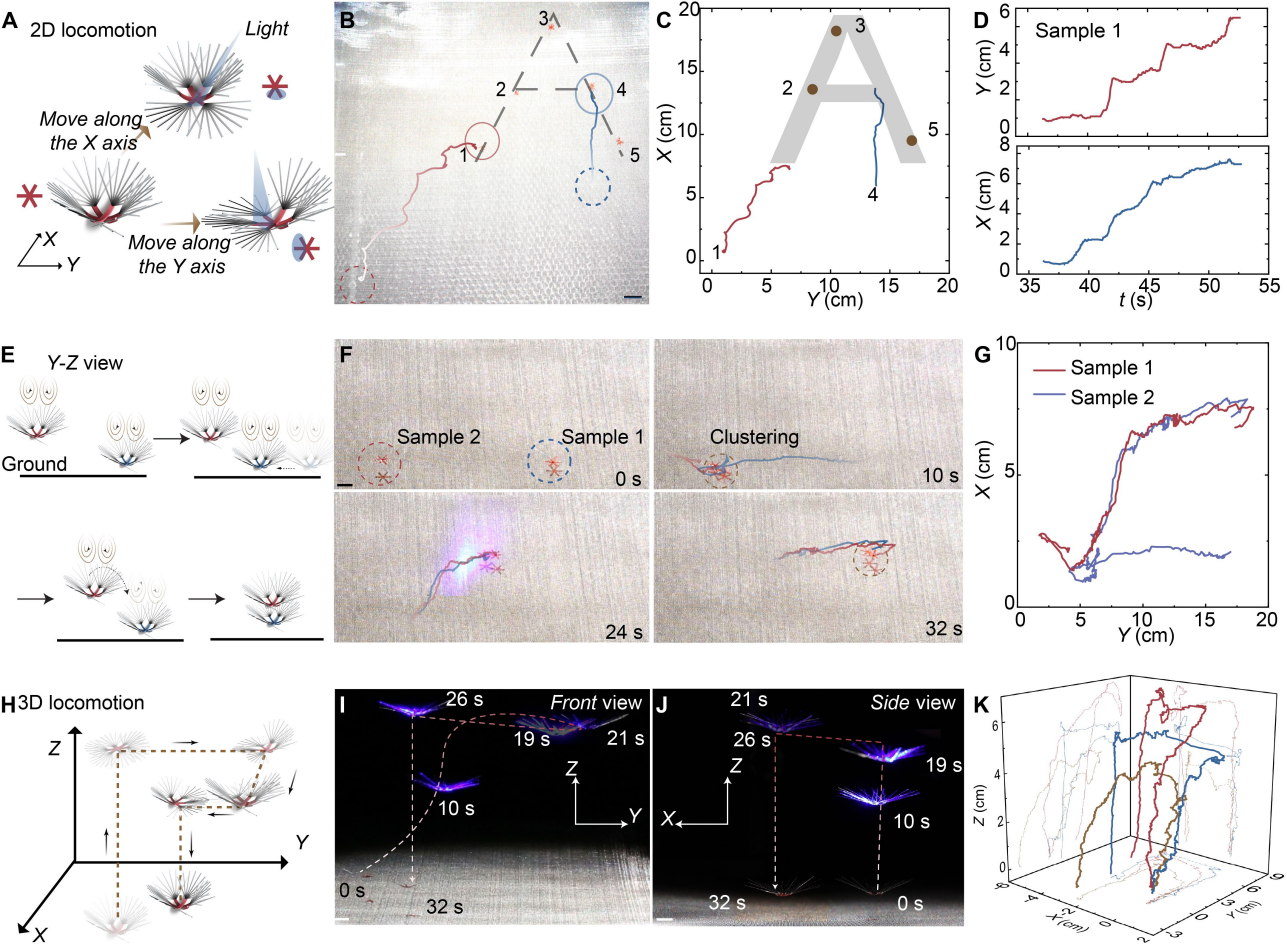
Agile maneuvering of the dandidrone. (**A**) Schematic of the light-controlled 2D locomotion in the horizontal *X*-*Y* plane. (**B**) Image of five dandidrones steered on the *X*-*Y* plane to reach the vertices of the letter “A.” (**C**) Trajectory of two dandidrones steered to form the “A” shape. (**D**) Time histories of the *Y* (top) and *X* (bottom) coordinates of sample 1. (**E**) Schematic drawing of the clustering between two dandidrones. (**F**) Snapshots of dandidrone pairs controlled by light. (**G**) Trajectory of two dandidrones in the *X*-*Y* plane. (**H**) Schematic of the 3D trajectory of the dandidrone. Superimposed images of the dandidrone performing light-controlled 3D locomotion from the (**I**) front view and (**J**) side view. (**K**) 3D trajectory of the dandidrone with projections onto the *X*-*Y*, *X*-*Z*, and *Y*-*Z* planes. Wind tunnel speed: 0.6 m s^−1^. Light intensity: 700 mW cm^−2^. Scale bars, 0.5 cm.

Whereas the above 1D maneuvers concern only the vertical direction, 2D maneuvers on the horizontal *X*-*Y* plane are presented in the following. Uneven light excitations between arm segments introduce an asymmetric deformation. This allows targeting any position in the horizontal plane, as schematically illustrated in fig. S46. Specifically, selective illumination of the left or right three-legged segments results in translational motion toward right or left, whereas targeting the front or rear segments enables movement backward or forward (Fig. 4A). The light spot position was manually controlled following the flier navigation, during which the light intensity the flier receiving is dynamic. The horizonal flight needs to be activated by an excitation above the intensity threshold upon the light onset (figs. S38 and S40), followed by a continuous flight dictated by the translational speed of the light spot (fig. S47). The flier velocity increases with the scanning speed of the light source. Noticed that, during the horizontal translocation, the flier has high stability in both orientation (nonspinning) and vertical position (fig. S48). The fine-tuning of the 2D positioning among five individual dandidrones to form the shape of the letter “A” is demonstrated in Fig. 4 (B and C) and movie S6. Airborne patterns forming the letters “A,”, “B,” and “C” are reported in Fig. 4D and figs. S49 to S51.

Mutual attraction was observed when two dandidrones are at a distance lower than 3 cm, which is equivalent to one dandidrone’s diameter. After interacting, two dandidrones form a cohesive unit that can subsequently be controlled like a single unit (Fig. 4, E and F, and movie S7). This cluster exhibits approximately the same terminal velocity and stability as an individual unit. Owing to the shift in the overall center of mass after aggregation, the cluster exhibits a characteristic orbital motion in the horizontal plane with slow speed about 0.47 cm s^−1^ (fig. S52). Despite this lateral revolution, the vertical (*Z*-direction) positional fluctuations and terminal velocity remain comparable to those of an individual flier, indicating preserved flight stability (fig. S53). Notably, the trajectory can be controlled by manipulating just one dandidrone inside the cluster, thereby enabling synchronized motion of the swarm (Fig. 4G and fig. S54). The 2D trajectory control of swarms of three dandidrones and the stability during the flight are reported in figs. S55 to S57.

Last, complex 3D trajectories are demonstrated, where the dandidrone is steered in different horizontal directions by choosing which of the six robotics arms is actioned (Fig. 4H). By manual handling of an optical fiber head inside the wind tunnel, a dandidrone is steered to ascend to a predefined height, perform a smooth lateral translation, and subsequently descend near its original position (Fig. 4, I and J, and movie S8). The 3D steering with trajectory control across three distinct target altitudes is shown by the representative paths in Fig. 4K (figs. S58 and S59).

## DISCUSSION

Previous dandelion-inspired photoresponsive fliers primarily rely on globally symmetric deformation of filamentary pappus structures, which allows tuning of the drag and terminal velocity but restricts movements to the vertical direction (*30*). This work introduces a discrete, multiarm architecture with independently actuated LCE muscles, which enable programmable asymmetric deformation, allowing 2D and 3D flight control that maintains the high level of flight stability. Rather than invoking a new aerodynamic mechanism, this design provides a means to actively manipulate the wake structure, enabling the formation of a stable and steady wake while independently controlling vertical and horizontal aerodynamic forces. Although we have achieved an agile maneuvering over the 3D space (Fig. 4, I to K), asymmetric deformation of the dandidrone inevitably results in vertical drift (figs. S60 and S61). This is because asymmetric deformation alters the drag, leading to either an upward or downward movement. This reduces the capacity for 3D steering (Fig. 4, H to J) along a precise trajectory.

Integrating a stimulus-response material with passive flight mechanisms represents an effective approach to control the flight of insect-scale fliers that are passively transported and distributed by the wind (*39*). We compared our actuator with representative driving systems recently reported for flying robots (table S1). For active fliers—the mechanical movement must produce sufficient lift to overcome gravity—an actuator with high power density is required. For passive fliers, because the wind energy becomes the driving force to sustain the motion in the mid-air, an actuator only needs to provide the minimal shape-morphing to induce the change of geometry, consequently triggering the translocation. It is worth noting that, optical actuation offers unique advantages in robotic flight, such as untethered control and wireless powering. Using light beam eliminates the need for onboard power supplies or complicated electronic circuits, which offer the exciting opportunities for system minimization through microfabrication of actuators with ever decreasing sizes. In this study, light stimulation has been selected as the primary actuation method due to that well-established applicability. However, this approach can be extended to any external stimulus (e.g., humidity and heat) capable of promoting the change in morphology (*40*, *41*), potentially unlocking a wide range of capabilities and operations. Furthermore, fully autonomous tasks would be possible if a sufficiently light energy source could be carried without excessively increasing the terminal velocity, limiting the endurance. Dandidrones equipped with sensing and communication capabilities have already been demonstrated (*23*), and current efforts are exploring biodegradable and self-degrading variants to eliminate the need for post–mission retrieval. The precise maneuverability shown in this study expands the potential applications of these systems, including environmental monitoring, surveillance, and target recognition. One particularly promising area is the monitoring of climate change. The 2021 Global Climate Observing System Status Report (*42*) identified gaps in existing observing systems that hinder an in-depth understanding of climate change and the identification of effective mitigation actions. The report highlights the critical need for increasing sensitivity, resolution, and coverage of in situ measurements. Whereas conventional sensing infrastructure remains spatially constrained and autonomous drones offer limited operational persistence, dandidrones—when outfitted with onboard sensors and deployed in swarms—could provide scalable, high-resolution, and long-duration in situ monitoring. Their silent operation, low ecological impact, and ease of deployment make them especially well-suited for sensitive or remote environments (*43*).

Furthermore, their demonstrated steerability enables complex, coordinated aerial behaviors, offering a powerful platform for distributed sensing systems. Environmental monitoring using passive microfliers can be enabled through the integration of lightweight functional devices. One promising strategy is the use of polymer-based, power-free sensors that directly respond to environmental stimuli such as humidity, pH, or light, through changes in color fluorescent or shape (*27*, *28*), eliminating the need for onboard power or electronics. In parallel, recent progress in soft electronics has made it possible to incorporate ultralight, flexible miniaturized sensors at the scale of less than 10 mg (*29*). These lightweight components could be mechanically and aerodynamically compatible with passive fliers, enabling distributed sensing without compromising flight performance. Further study is necessary to achieve advanced steerability in nonuniform or time-varying flows, where airflow fluctuations are expected to influence instantaneous trajectories. At present, controllability under turbulent conditions is anticipated to persist in a statistical sense, allowing for regulation of averaged trajectories over characteristic timescales. Future work will extend this platform to dynamic flow conditions, including variable wind speeds and imposed turbulence, to evaluate robustness and applicability in realistic outdoor scenarios. Building on the demonstrated material-level feedback mechanisms for vertical motion regulation, future developments will incorporate feedback-assisted control in the horizontal plane, improving adaptivity under dynamically varying environmental conditions. The passive, lightweight design and rapid material response suggest potential resilience to moderate flow fluctuations, enabling operation in complex environments. Although additional development is required before large-scale deployment becomes feasible, this work lays the foundation for soft, untethered, and environmentally adaptive microfliers that have the potential to transform how we observe and interact with the natural world (*44*).

In natural environments, the passive flight is intrinsically stochastic due to turbulent airflows and environmental fluctuations. Rather than attempting to optimize the positional control as performed inside a stabilized wind tunnel, the control strategy for outdoor experiments would be designed to regulate its key flight events, i.e., liftoff, descent, and landing, within a probabilistic process. For instance, future applications may be foreseen to be the sunlight or humidity from a lake surface can drive the takeoff/landing of the flier, onto some premarked environmental locations. Recent advances in low-power-driven LCEs (*41*) and ultrasensitive films (*45*) responding to humidity make such science fiction closer to reality.

To conclude, drawing inspiration from the aerodynamics of dandelion diaspores, we developed ultralight hexapodal dandidrones that harness photomechanical LCEs to achieve unprecedented control and maneuverability. They have a weight of the order of 1 mg, similar to their natural counterpart, and a range of terminal velocities from 88 to 146% of the mean terminal velocity of the dandelion diaspore. The shape morphing of the hexapodal structure creates asymmetric, SVRs and a lateral force, enabling the control of their horizontal displacement. In wind tunnel experiments, dandidrones demonstrated superior linear and rotational stability compared to the natural dandelion. The analysis of the flow field reveals high aerodynamic stability in every operational configuration. We have demonstrated the control of the height inside an upward free airflow, light-induced body flipping, formation of 2D patterns, swarming, and navigation through complex 3D trajectories. This study simplifies structural design, reduces weight, and enhances energy efficiency, expanding the potential applications of microfliers for long-range and long-duration sensing missions.

## MATERIALS AND METHODS

### Materials

1,4-Bis-[4-(6-acryloyloxyhexyloxy)benzoyloxy]-2-methylbenzene (99%, RM82) was obtained from SYNTHON Chemicals. 6-Amino-1-hexanol and dodecylamine were obtained from TCI, 2,2-dimethoxy-2-phenylacetophenone was obtained from Sigma-Aldrich, and Disperse Red 1 and Disperse Blue 14 were obtained from Merck. All chemicals were used as received.

### Fabrication of the LCE film

Liquid crystal cells were prepared by assembling two coated glass substrates. One substrate was coated with polyvinyl alcohol (PVA; 5 wt % in water, spin coated at 3000 rpm for 1 min and baked at 90°C for 10 min) for uniaxial alignment, whereas the other was coated with polyimide (PI; spin coated at 3000 rpm for 1 min and baked at 180°C for 20 min) to achieve homeotropic alignment. A 5-μm microsphere spacer (Thermo Fisher Scientific) was used to control the gap between the glass slides, determining the thickness of the LCE film. The liquid crystal mixture, consisting of 0.3 mmol of RM82, 0.115 mmol of 6-amino-1-octanol, 0.115 mmol of dodecylamine, and 2.5 wt % Irgacure 651, was melted at 85°C and infiltrated into the cell via capillary action. The cell was held at 85°C for 10 min and then cooled to 63°C at a rate of 1°C min^−1^ and stored at 63°C for 24 hours to facilitate the aza-Michael addition reaction (oligomerization). Polymerization was completed by ultraviolet irradiation (365 nm, 180 mW cm^−2^ for 10 min). After opening the cells with a razor blade, 1 mg of Disperse Red 1 was spread on the sample surface and diffused into the elastomer on a hot plate at 100°C for 10 min.

### Sample assembly

Three LCE strips were bonded together to form a hexapodal structure. Each pappus was composed of nine filaments adhered together. The dimensions of the LCE strips were 4 mm by 2 mm by 0.05 mm, with the combined mass of the 54 filaments being 0.66 mg, whereas the LCE strips themselves weighed 0.54 mg. The total mass of the dandidrone is 1.20 mg.

### Power density and energy conversion efficiency estimation

The power density of the LCE actuator was defined as

$$\text{power density}=\frac{m_{\text{load}}g∆h}{∆tm_{a}}$$

where $m_{\text{load}}$ = 15 mg is the mass of the load attached to the actuator, ∆h = 0.25 cm is the lifting height, *g* = 9.8 m s^−2^ is the gravitational acceleration, ∆*t* = 1.1 s is the actuation time, and $m_{a}$ = 3 mg is the mass of the LCE actuator.

The energy conversion efficiency of the LCE actuator was defined as

$$\text{energy conversion efficiency}=\frac{m_{\text{load}}g∆h}{∆tP_{a}}$$

where $P_{a}$ is the incident optical power on the LCE actuator.

### Stimuli sources

A collimated beam from a continuous-wave solid-state laser (ROITHNER, 2 W, 457 nm) was directed onto the LCE to induce deformation. The absorbed optical power was calculated as the product of the light intensity (*I*) and the illuminated area.

### Drop test

Drop tests were conducted by releasing dandidrones of varying sizes from a height of 1 m, repeating each trial 10 times. The descent was recorded using a digital single-lens reflex camera (Canon EOS 60D) operating at 25 frames/s. Trajectories were analyzed using the TRACKER software to determine the terminal velocity (*u*_t_), defined as the asymptotic speed at which weight and aerodynamic drag reach equilibrium. The mean terminal velocity between 10 repeats was reported. To further investigate the influence of mass on the terminal velocity, we attached calibrated plasticine weights to the dandidrones and repeated the measurements under identical conditions.

### Vertical wind tunnel

The tunnel is made of four main sections (fig. S30A): the test section, a section housing the honeycomb, the four-fan array, and the settling chamber. The tunnel is a part of a translating vertical wind tunnel setup but for the current experiments the tunnel was kept in a fixed position (*46*). All sections of the tunnel are separated by stainless steel woven wire meshes. The mesh immediately upstream of the test section has a 1.98-mm hole size and 0.559-mm-thick wires. The honeycomb is made of aluminum alloy with a density of 76.9 kg m^−3^ and cells that are 1/2 inches (1.27 cm) in size to suppress horizontal velocity components. Each of the four fans, which are controlled by an Arduino Uno, have a diameter of 0.12 m and a maximum angular velocity of 1050 rpm. Figure S30B presents a photograph of the laboratory setup. In the test section, the mean turbulence intensity *T*_u_ = *u*′/*u*_t_ is lower than 2%, where *u*′ is the root mean square of the vertical velocity fluctuations.

### Flow visualization

Visualization of the flow as seen in fig. S21 was obtained by overlaying raw PIV images acquired by a high-speed camera. Fifty instantaneous images were stacked for each case, using the ImageJ software, and their average was calculated. The images were further adjusted for brightness and contrast.

### Particle image velocimetry

Di-ethyl-hexyl-sebacate (DEHS) particles were spread from the bottom of the wind tunnel and a 1.5-W continuous-wavelength laser was used to illuminate the DEHS particles on a vertical plane of the test section. High-resolution images were captured with a Fastcam Photron camera equipped with a Tamron 180-mm F3.5 SP AF Di Macro Lens. High-speed image acquisition was performed at frame rates ranging from 500 to 1125 frames/s. The LaVision Davis 10 software was used for the processing of the images and the computation of velocity field, whereas MATLAB was used for subsequent postprocessing. Preprocessing included subtracting the minimum pixel intensity over 15 images to minimize noise. A multipass linear window deformation technique was applied, starting with an interrogation window of 64 × 64 pixels and progressively refining to a window of 32 × 32 pixels in subsequent passes. Subpixel displacements were determined using 2D Gaussian regression, with a 75% window overlap. The spatial resolution achieved was 1.55 mm, corresponding to a maximum displacement of 16 pixels at a flow velocity of 0.8 m s^−1^. The particle size averaged ∼3 pixels, and the seeding density was 0.025 particles/pixel. To prevent spurious vector generation near the model, geometric masking (polygonal type) was applied in Davis 10, followed by vector interpolation within masked regions. Vectors exceeding an SD threshold of 2 were replaced to ensure accuracy.

### Numerical methodology

The airflow around the hexapodal fliers has been numerically modeled using a porous medium approach. The computational model consists of a hexapodal core at the center, surrounded by a disk-shaped porous region representing the filaments. The core is modeled as a thin solid structure, whereas the porous disk is treated as a volume of fluid subjected to Darcy resistance. The core dimensions closely match those of experimental specimens, whereas the porous disk has an aspect ratio of 10 and a diameter (*d*) of 3 cm. The homogeneous and isotropic porous resistance is characterized by the Darcy number, *Da* = *K*/*d*^2^, where *K* is the permeability. Darcy numbers in this study range from *Da* = 10^−1^ to *Da* = 10^−6^, representing very coarse filaments (the porous disk exerts nearly no resistance) to very dense filaments (the porous disk is effectively impermeable), respectively. The 3D Navier-Stokes equations are directly solved in OpenFOAM, a finite volume–based, open-source software package, using the pimpleFoam solver. The Darcy resistance is incorporated as an explicit porosity source term in the momentum equation. Second-order accurate schemes are used for both temporal and spatial discretization, with residual tolerances of 10^−8^ for the velocity components and pressure to ensure numerical accuracy. The computational domain is cylindrical, with a diameter of 24*d*, and extends 15*d* and 25*d* upstream and downstream of the model, respectively. The computational domain is discretized using a highly refined unstructured mesh of polyhedral elements in total, with cells concentrated in the vicinity of the porous disk and core and expanding progressively toward the domain boundaries. A uniform inlet velocity matching the experimentally measured terminal velocity (*u*_t_ = 0.4 m s^−1^) is prescribed at the inlet. A zero-gradient boundary condition is applied at the outlet for velocity, with a fixed reference pressure. Free-slip and no-slip boundary conditions are applied at the side wall of the domain and the surface of the hexapodal core, respectively.

### Impact of the opening angle on the size of the SVR

To characterize the SVR, an axis (*X*′) through nodes and one axis (*Z*′) through the saddle points were introduced. Figure S24E schematically represents the wake structure, with points *N*_1_ and *N*_2_ marking nodes along line *X*′ and points *S*_1_ and *S*_2_ marking saddle points along line *Z*′.

## Supplementary Materials

### The PDF file includes:

Notes S1 to S4 Figs. S1 to S61 Table S1 Legends for movies S1 to S9 References
