# Supplementary material for: Optical maneuvering of dandelion-inspired fliers with vortex-enabled stability
Source: Sci Adv. 2026 Jul 29;12(31):eaee8014. doi: 10.1126/sciadv.aee8014 (PMC13418530; doi:10.1126/sciadv.aee8014)
Supplement: Supplementary file 1 — Notes S1 to S4 Figs. S1 to S61 Table S1 Legends for movies S1 to S9 References [file sciadv.aee8014_sm.pdf]

Science Advances
AAAS

Supplementary Materials for

Optical maneuvering of dandelion-inspired fliers with vortex-enabled stability

Jianfeng Yang *et al.*

Corresponding author: Hao Zeng, hao.zeng@tuni.fi; Ignazio Maria Viola, i.m.viola@ed.ac.uk

Sci. Adv. **12**, eaee8014 (2026)
DOI: 10.1126/sciadv.aee8014

The PDF file includes:

Notes S1 to S4
Figs. S1 to S61
Table S1
Legends for movies S1 to S9
References

Other Supplementary Material for this manuscript includes the following:

Movies S1 to S9

Supplementary Note S1: Design, Optimization, Mechanism, and Optical Properties of Dandidrones

The dandelion-inspired drone (dandidrone) is constructed by integrating liquid crystalline elastomer (LCE) strips with biomimetic filaments (figs. S1 and S2). To optimize flight stability and actuation efficiency, a six centro-aligned LCE arm configuration was adopted. Two/four-arm designs enabled basic take-off but were unstable in mid-air (fig. S3). Increasing arm number improved symmetry and stability (fig. S4), yet reduced spacing hindered independent actuation. Thus, the six-arm design provides the optimal compromise. These biomimetic filaments closely resemble natural dandelion filaments in both stiffness (figs. S5 and S6) and radius (fig. S17G, H), ensuring comparable aerodynamic performance. The LCE serves as the central active component, enabling mechanical actuation through a thermally induced nematic-to-isotropic phase transition (figs. S7 and S8). To achieve light responsiveness, Disperse Red 1 (DR1) is incorporated into the LCE matrix, allowing precise curvature control by modulating illumination intensity (figs. S9 and S10). The generated bending forces, ranging from 0.5 mN to 3 mN, are sufficient to support the weight of the filaments (0.00098 mN) (fig. S11) and the aerodynamic force. The LCE actuator exhibits a response time of approximately ~1 s (fig. S12), a power density 0.11 W kg^{-1}, and energy conversion efficiency on the order of $\sim 3 \times 10^{-6}$ (fig. S13). Additionally, photomechanical durability tests demonstrate stable performance over 100 actuation cycles, with no observable material degradation within the experimental timeframe (fig. S14). All these establish a foundation for photothermally induced shape-morphing of dandidrone (fig. S15), with wind tunnel experiments confirming that photothermal actuation remains largely unaffected by the wind speed, ensuring robust operation in dynamic airflow conditions (fig. S16).

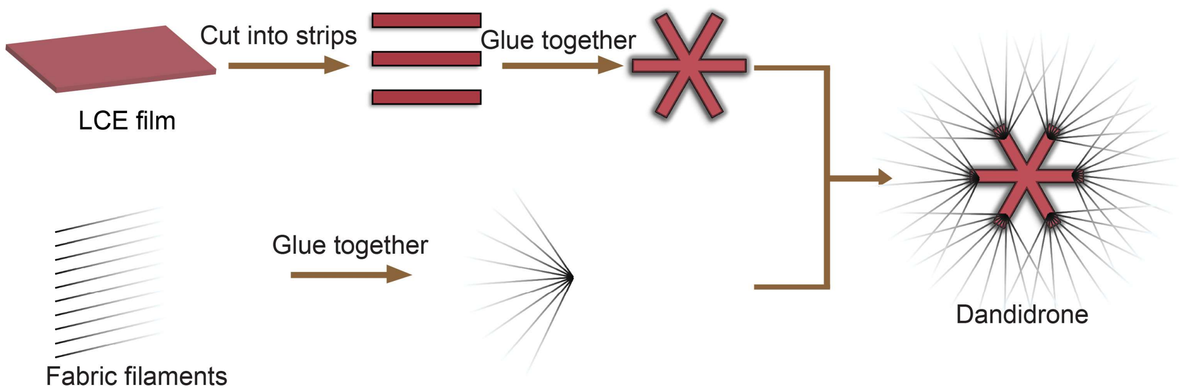

Fig. S1. The fabrication process of the dandidrone. Schematic illustration of the fabrication process of the dandidrone.

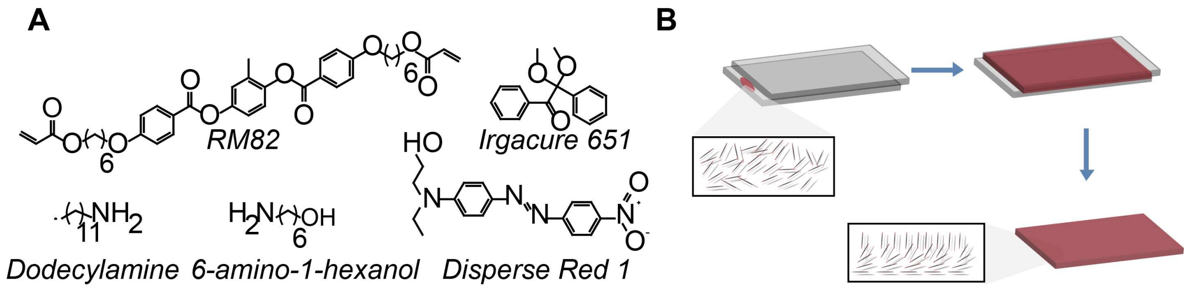

Fig. S2. Synthetic steps of the soft actuator. (**A**) Chemical structures of all molecules in use. Pre-cured mixture contains 0.3 mmol RM82, 0.115 mmol 6-amino-1-octanol, 0.115 mmol dodecylamine and 2.5 wt% Irgacure 651. (**B**) Schematic drawing of the LCE film preparation process and molecular alignment.

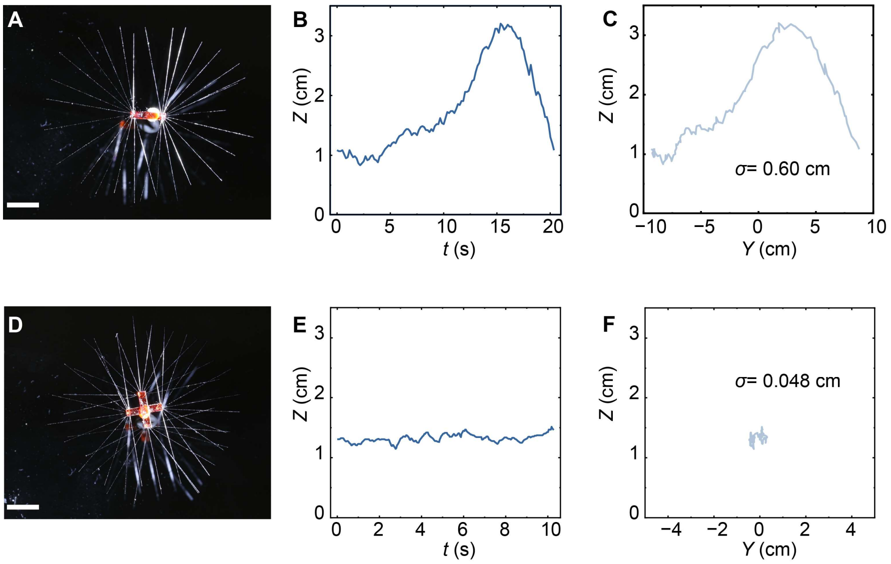

Fig. S3. The stability of the dandidrone with two and four arms. (**A**) Photographs of a dandidrone with two arms. (**B**) The vertical coordinate of dandidrone with two arms over time. (**C**) The *Y* and *Z* coordinates of dandidrone with two arms over 1 s (25 data points). (**D**) Photographs of a dandidrone with four arms. (**E**) The vertical coordinate of dandidrone with four arms over time. (**F**) The *Y* and *Z* coordinates of dandidrone with four arms over 1 s (25 data points). Wind tunnel speed: 0.6 m s^{-1}. All the scale bars are 5 mm.

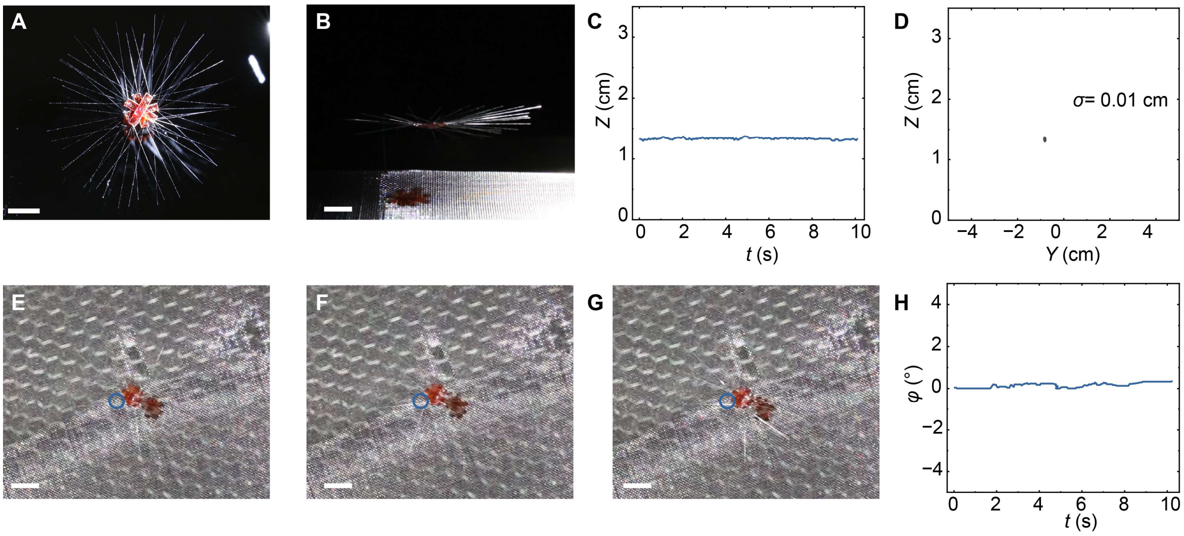

Fig. S4. The stability of the dandidrone with eight arms. (**A**) Photographs of a dandidrone with eight arms. (**B**) Photographs of a dandidrone with eight arms floating in the wind tunnel. (**C**) The vertical coordinate of dandidrone with eight arms over time. (**D**) The *Y* and *Z* coordinates of dandidrone with eight arms over 1 s (25 data points). (**E-G**) Snapshots of the dandidrone with eight arms. (**H**) The change in rotation angle φ of the dandidrone with eight arms with time. Wind tunnel speed: 0.6 m s^{-1}. All the scale bars are 5 mm.

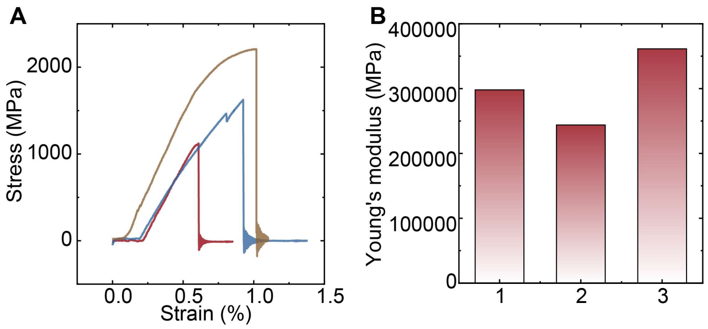

Fig. S5. Mechanical properties of biomimetic filaments. (**A**) Stress-strain diagram and (**B**) Young’s modulus of fabric filaments for three different samples.

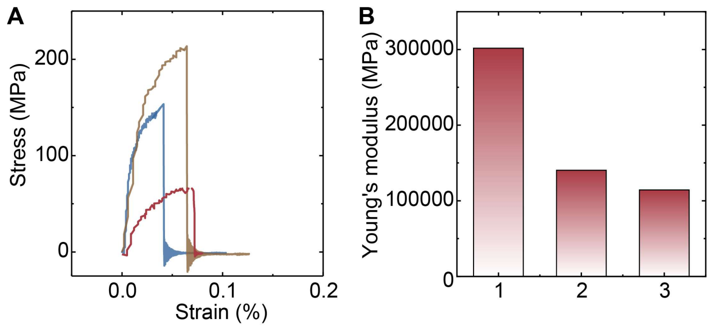

Fig. S6. Mechanical properties of natural dandelion filaments. (**A**) Stress-strain diagram and (**B**) Young's modulus of dandelion filaments for three different samples.

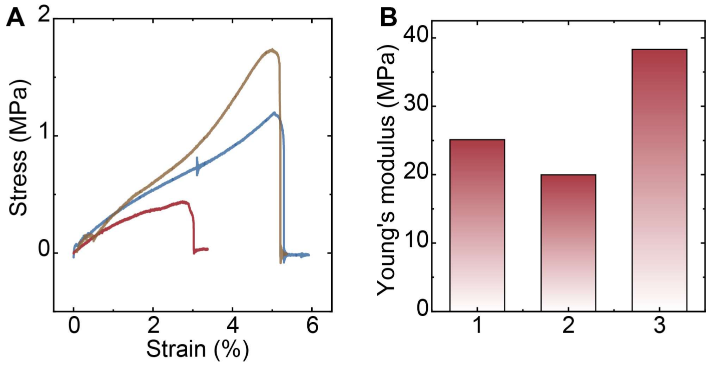

Fig. S7. Mechanical properties of the LCE film. (**A**) Stress-strain diagram and (**B**) Young's modulus of LCE strips for three different samples.

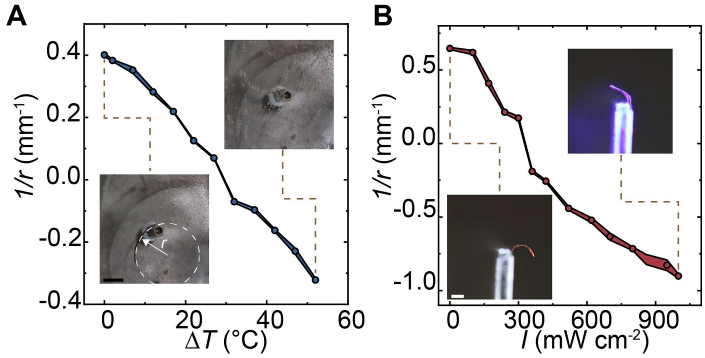

Fig. S8. Thermally and optically induced deformations in LCE film. (**A**) Curvature (1/*r*, with *r* the radius of curvature) of the LCE strip at different temperatures. Inset: images of the LCE strip at various temperatures. The sample was heated in a temperature-controlled water bath. (**B**) Change in curvature of the LCE strip under different light intensities. Inset: images of the LCE strip exposed to varying light intensities. All scale bars are 5 mm. The error bars are displayed as mean values +/- standard deviation (n = 3). The same sample was measured repeatedly.

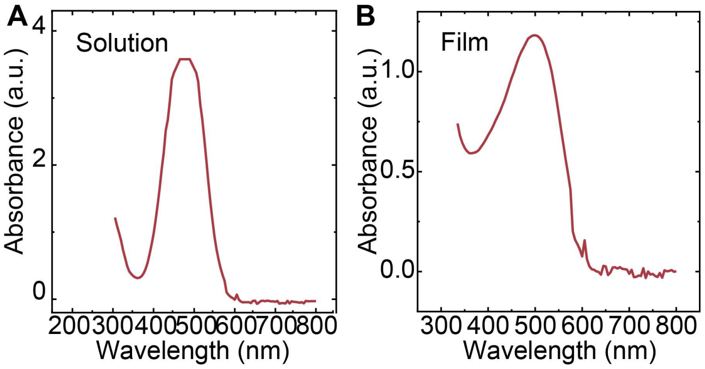

Fig. S9. Optical property of the LCE film. Absorption spectrum of (**A**) the dye solution and (**B**) the polymer film after dyeing. Film thickness: 5 μm.

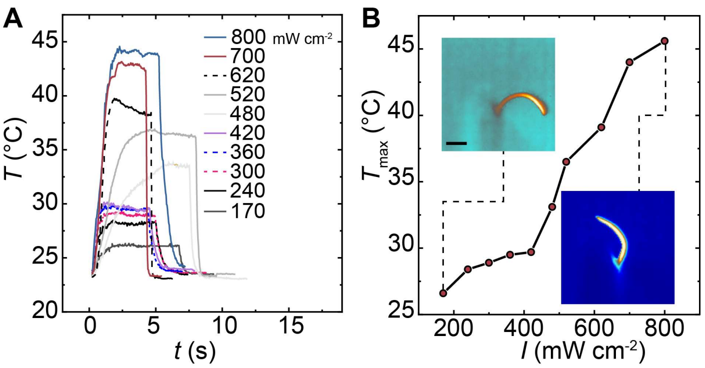

Fig. S10. The photothermal kinetics of the LCE film. (**A**) The time-history of the temperature during the bending of the LCE film under different light intensities. (**B**) The maximum temperature ($T_{\max}$) achieved under various light intensities. Inset: Infrared image of the LCE film at different light intensities. The scale bar is 5 mm. The dimensions of the LCE film: 15 mm × 3 mm × 0.05 mm.

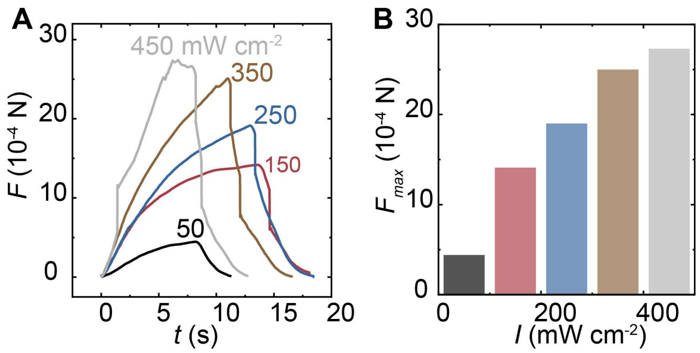

Fig. S11. Light-activated forces of the LCE strip. (**A**) Time history of the blocking force of a straight LCE strip under different light illuminations. (**B**) The maximum blocking force of an LCE film upon different light intensities. Strip size for material characterization: 0.7 cm × 0.2 cm × 0.005 cm.

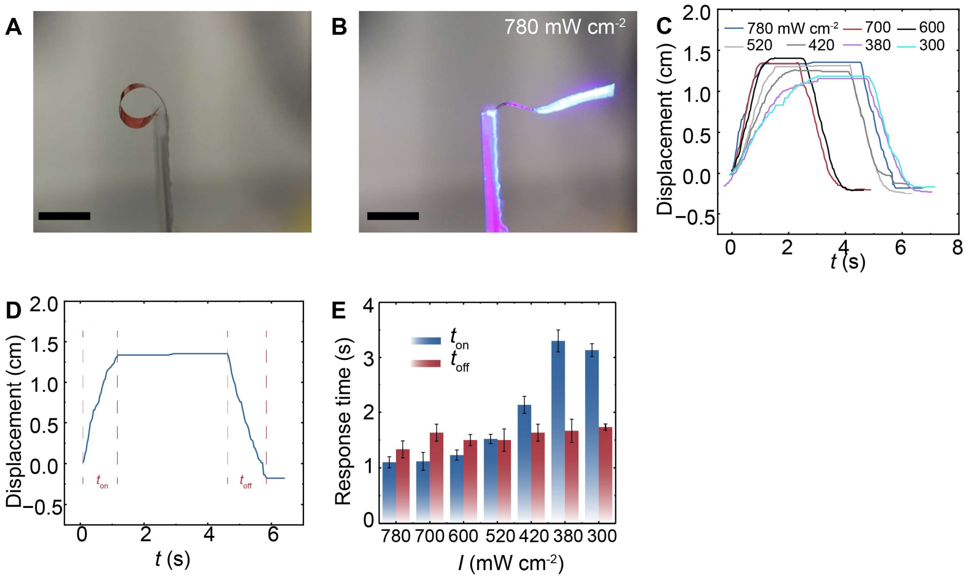

Fig. S12. Response time of the LCE actuator. (**A**) Photograph of the LCE in the absence of illumination. (**B**) Photograph showing LCE deformation under light irradiation. (**C**) Actuation kinetics under different illumination intensities. (**D**) Definition of the response times for light-induced bending (t_{on}) and subsequent relaxation (t_{off}). (**E**) Response times under different illumination intensities. Dimension of LCE: 1 cm × 0.3 cm × 0.05 cm. The error bars indicate *s.d.* for n = 3 measurements. All scale bars are 5 mm.

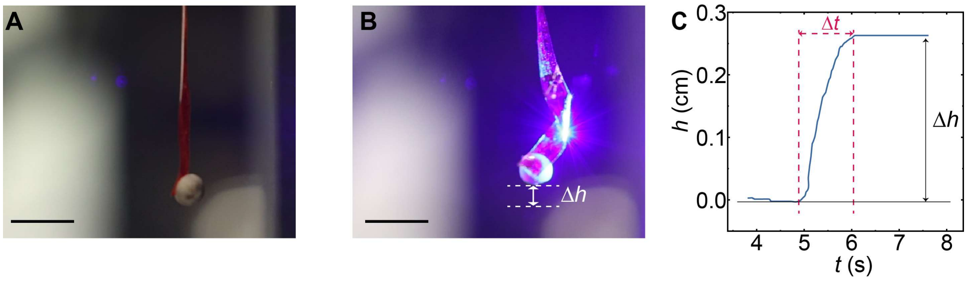

Fig. S13. Energy efficiency of the LCE actuator. (**A**) The LCE actuator suspending weight in the absence of illumination. (**B**) The LCE actuator lifting weight under light irradiation. (**C**) Trajectory of the LCE actuator lifting weight under light irradiation. Light intensity: 900 mW cm^{-2}. Light spot size: 3 mm. LCE actuator size: 12 mm × 2 mm × 0.05 mm. All scale bars are 5 mm.

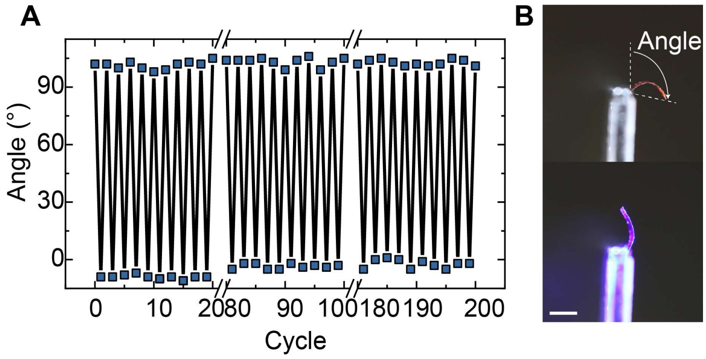

Fig. S14. Cycle test of an LCE strip. (**A**) Bending angle of an LCE strip during one hundred light actuation cycles. Light: 460 nm, 400 mW cm^{-2}. (**B**) Photographs of light-induced deformation of an LCE strip. The image is identical to that shown in fig. S8B and is reused here to illustrate the deformation of the LCE strip before and after light irradiation. The scale bar is 5 mm.

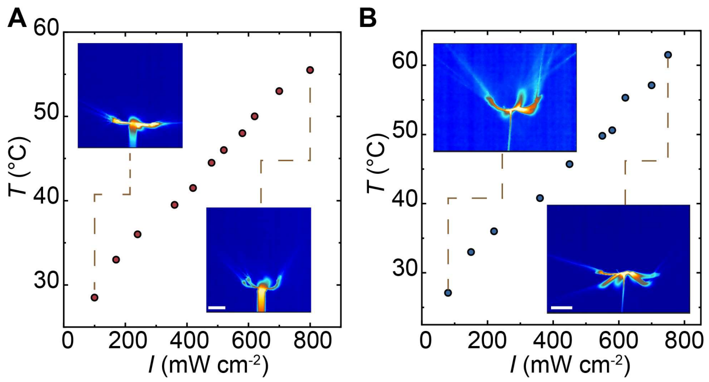

Fig. S15. The photothermal property of dandidrones. The temperature of the dandidrone with an initially (**A**) open and (**B**) closed configuration upon different light intensities. Insets: Infrared photographs of the dandidrone with an initially (**A**) open and (**B**) closed configuration for two different light intensities. All scale bars: 5 mm.

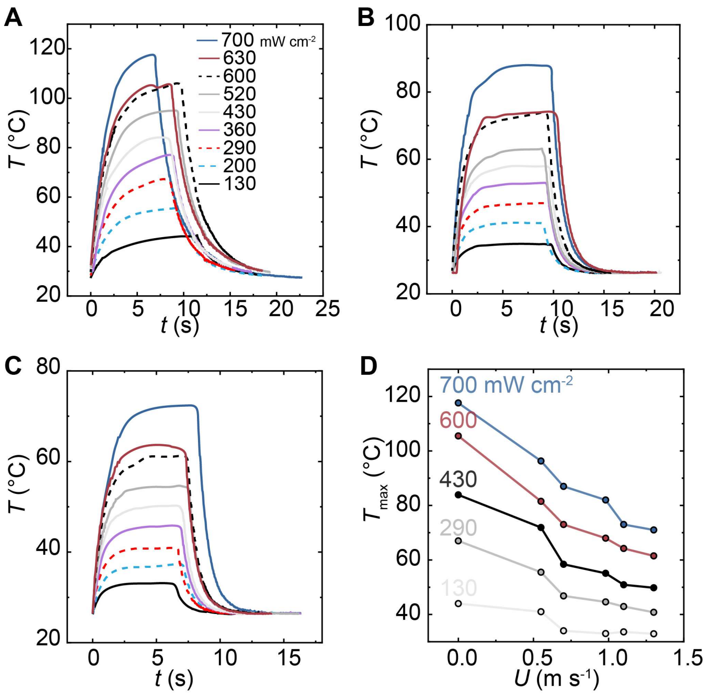

Fig. S16. The photothermal property of dandidrones in different airflows. Time-history of the temperature of the LCE film bending under varying light intensities in an airflow speed of (**A**) 0 m s^{-1}, (**B**) 0.7 m s^{-1}, and (**C**) 1.3 m s^{-1}. (**D**) The maximum elevated temperature $T_{\max}$ of the LCE film at different flow speeds under different light intensities. The dimensions of the LCE film: 5 mm × 2 mm × 0.05 mm.

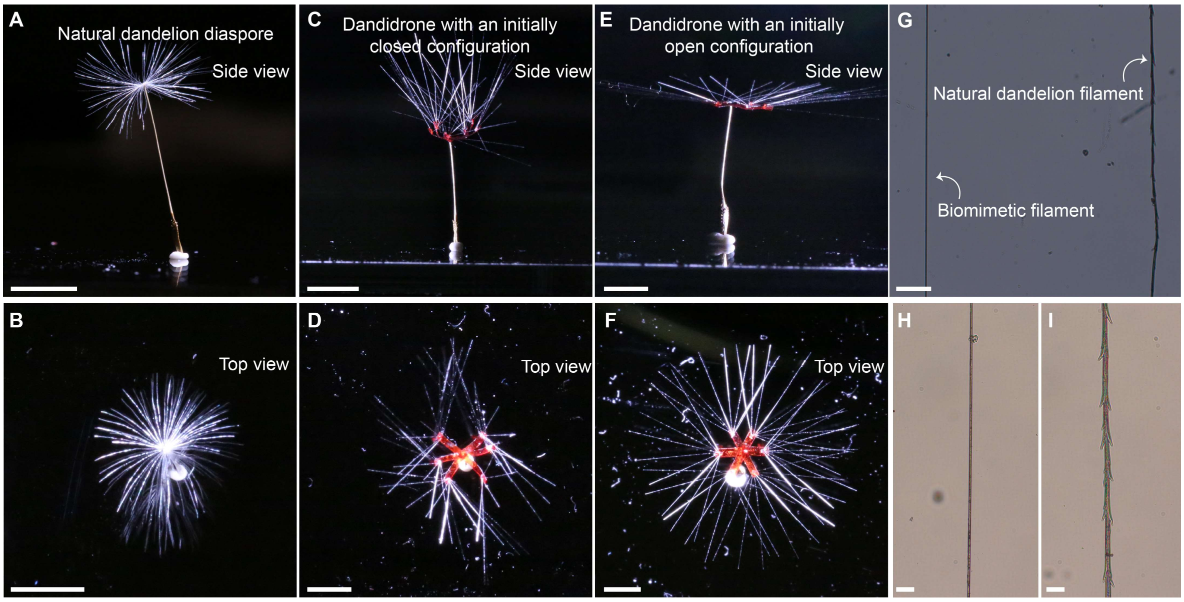

Fig. S17. Photographs of the natural dandelion diaspore and the dandidrones. (**A**) Side-view and (**B**) top-view of natural dandelion diaspores. (**C**) Side-view and (**D**) top-views of the dandidrone with an initially closed configuration. (**E**) Side-view and (**F**) top-view of the dandidrone with an initially open configuration. All the scale bars in (**A-F**) are 5 mm. (**G**) The microscopy images of the biomimetic and natural filaments. The scale bar is 500 µm. The zoomed-in view of the microscopy image of the biomimetic (**H**) and natural filament (**I**). The scale bars in (**H, I**) are 100 µm.

Supplementary Note S2: Stability of the Separated Vortex Ring

The number of filaments controls the permeability of the dandidrone and thus its descent stability. CFD simulations demonstrate that wake stability depends on permeability, quantified by the Darcy number (*Da*) (fig. S18). Both highly permeable ($Da = 1\times10^{-1}$) and nearly impermeable ($Da = 1\times10^{-6}$) cases produce strong shear layers, asymmetric wakes, and vortex shedding, resulting in unstable motion. In contrast, an intermediate permeability ($Da = 1\times10^{-3}$) yields symmetric streamlines and a stable vortex ring, leading to balanced forces and steady descent. Iso-surfaces of Q-criterion confirm that only structures within this optimal permeability range exhibit stable SVRs (fig. S19). Experiments also show that too few filaments (n = 18) or too many (n = 150) leads to irregular or chaotic falling due to insufficient or excessive vortex shedding, whereas an intermediate range (n = 30-54) enables steady descent (fig. S20). The separated vortex ring (SVR) is a toroidal vortex in the wake of the dandidrone. Its ad hoc porous structure allows the SVR to occur for any opening angle and for both symmetric and asymmetric configurations (fig. S21). Although Mie-scattering images near the centre may be partially obscured due to laser reflections from the flier surfaces, PIV analysis still provides reliable velocity fields and clearly identifies a stable SVR. Corresponding CFD results further confirm the presence of the SVR and show that the wake remains steady even in regions close to the dandidrone (fig. S22). Additional 3D simulations using iso-surfaces of Q-criterion demonstrate that, even in asymmetric configurations, the wake remains stable with a persistent SVR, without vortex shedding, and with a constant aerodynamic pitching moment acting on the flier (figs. S22 and S23, movie S9).

The SVR is a stable vortex, viz. the flow field forming the SVR has a constant topology at any instant (a toroidal shape with two saddles and two nodes, see fig. S24). If the filamentous structure of the dandidrone was impervious, the vortex would be unstable, resulting in a von Kármán vortex street (Fig. 2I-2L). Instead, the wake of the dandidrone shows a stable SVR both at small and large opening angles (fig. 25).

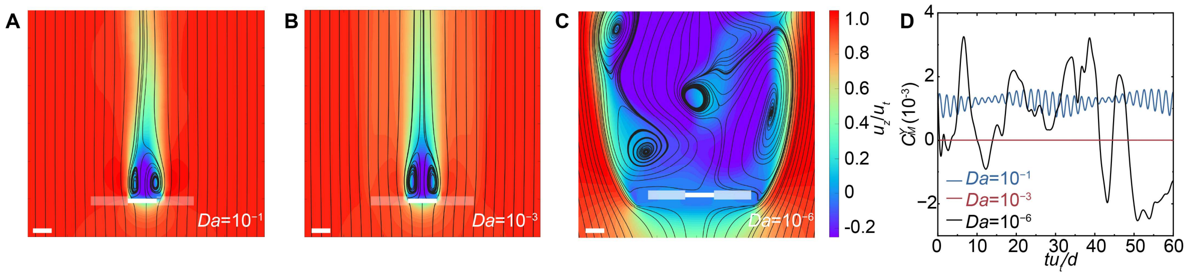

Fig. S18. CFD simulation for symmetric dandidrone. Instantaneous flow fields and streamlines from CFD simulations of (**A**) the hexapod with few filaments (Da = 10^{-1}), (**B**) symmetric dandidrone with optimal number of filaments (Da = 10^{-3}), and (**C**) effectively impervious dandidrone with large number of filaments (Da = 10^{-6}). (**D**) Time series of aerodynamic pitching moments (C_M^Y) on the fliers. The moment coefficient (C_M) for the stationary numerical simulations is defined as $C_M = \frac{M}{\bar{F}_D d}$, where M is the moment about the centre of the model, $\bar{F}_D$ is the mean drag force computed over the quasi-steady period. Scale bars are 0.5 cm.

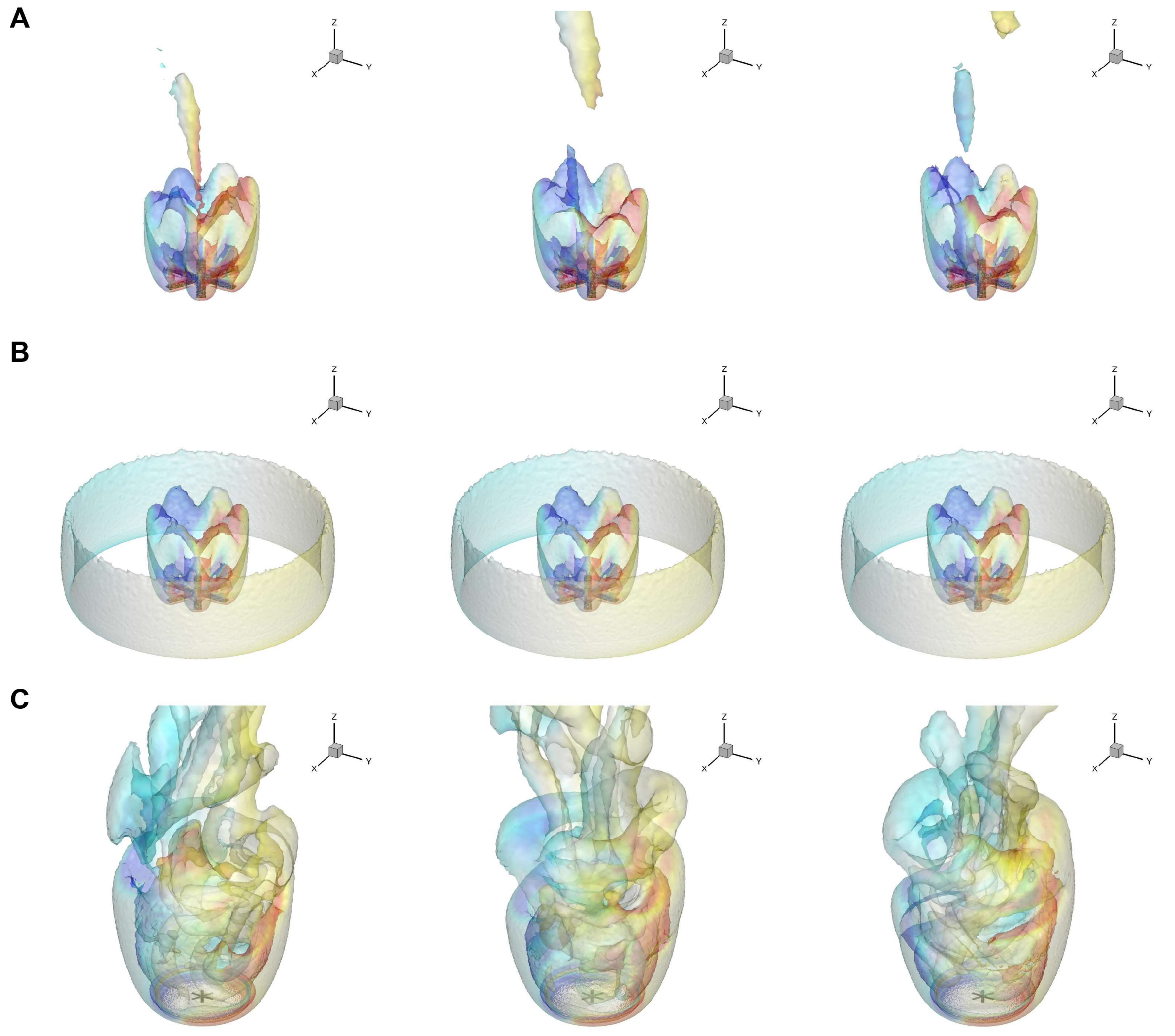

Fig. S19. 3D wake computation. Consecutive snapshots of isosurfaces of Q-criterion (Qd^2/u_t^2) coloured by vorticity ($\omega_x d/t$), ranging from −5 (blue) to +5 (red), from high-fidelity CFD simulations exhibiting (**A**) an unsteady periodic wake at $Da= 10^{-1}$, (**B**) a steady wake at $Da=10^{-3}$, (**C**) a highly unsteady wake with vortex shedding at $Da=10^{-6}$. u_t: terminal velocity. d: structural diameter. ω_x: vorticity.

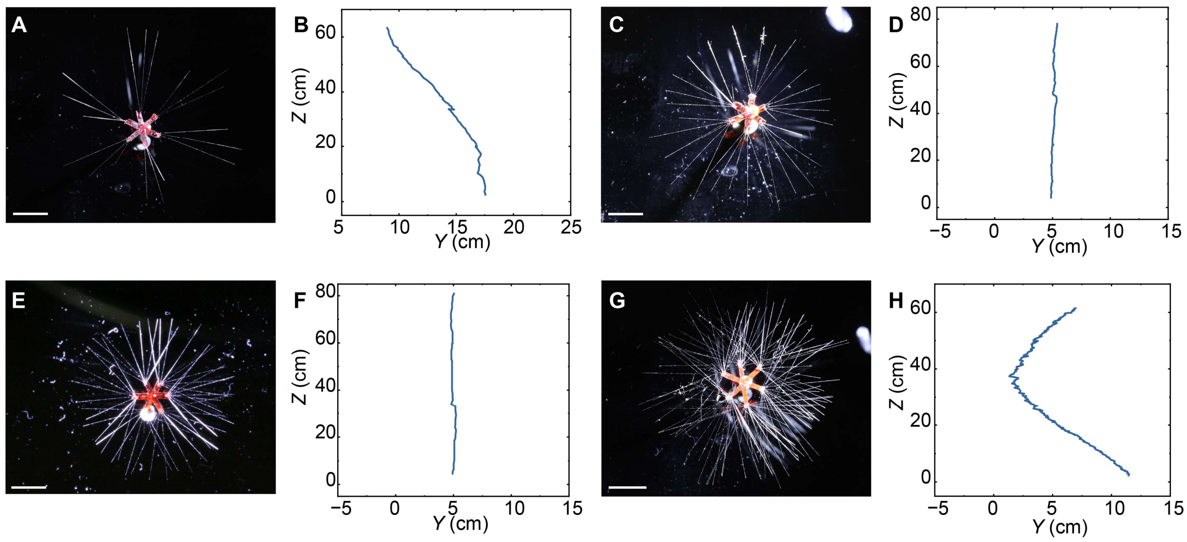

Fig. S20. Free-fall experiments of dandidrones with different filament numbers. (**A**) Photographs of dandidrone with 18 filaments. (**B**) Free-fall trajectory of the dandidrone with 18 filaments. (**C**) Photographs of dandidrone with 30 filaments. (**D**) Free-fall trajectory of the dandidrone with 30 filaments. (**E**) Photographs of dandidrone with 54 filaments. The image is identical to that shown in fig. S17F and is reused here to maintain consistency in representing the top-view of the six-legged dandidrone. (**F**) Free-fall trajectory of the dandidrone with 54 filaments. (**G**) Photographs of dandidrone with 150 filaments. (**H**) Free-fall trajectory of the dandidrone with 150 filaments. All scale bars are 5 mm.

A B C

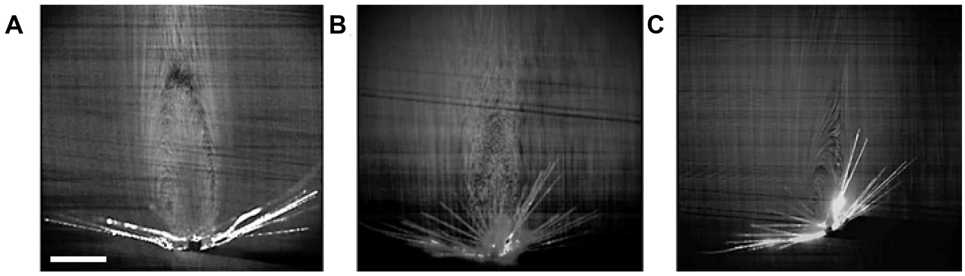

Fig. S21. The SVR at different opening angles. Photographs of the separated vortex ring in the wake of a dandidrone with an opening angle of 180° (**A**) and 150° (**B**), and an asymmetric opening angle of 120° (**C**). The scale is 5 mm.

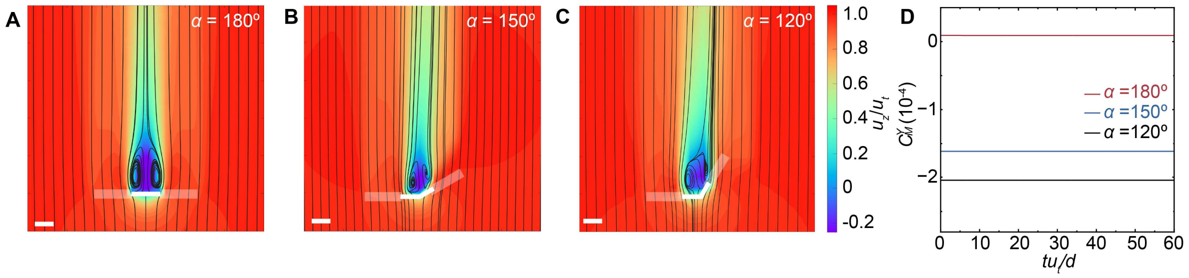

Fig. S22. **CFD simulation for asymmetric dandidrone.** Instantaneous flow fields and streamlines from CFD simulations of (**A**) symmetric dandidrone with opening angle of 180°, (**B**) an asymmetric opening angle of 150°, and (**C**) an asymmetric opening angle of 120°. (**D**) Time series of the aerodynamic pitching moments (C_M^Y) on the fliers. Scale bars are 5 mm.

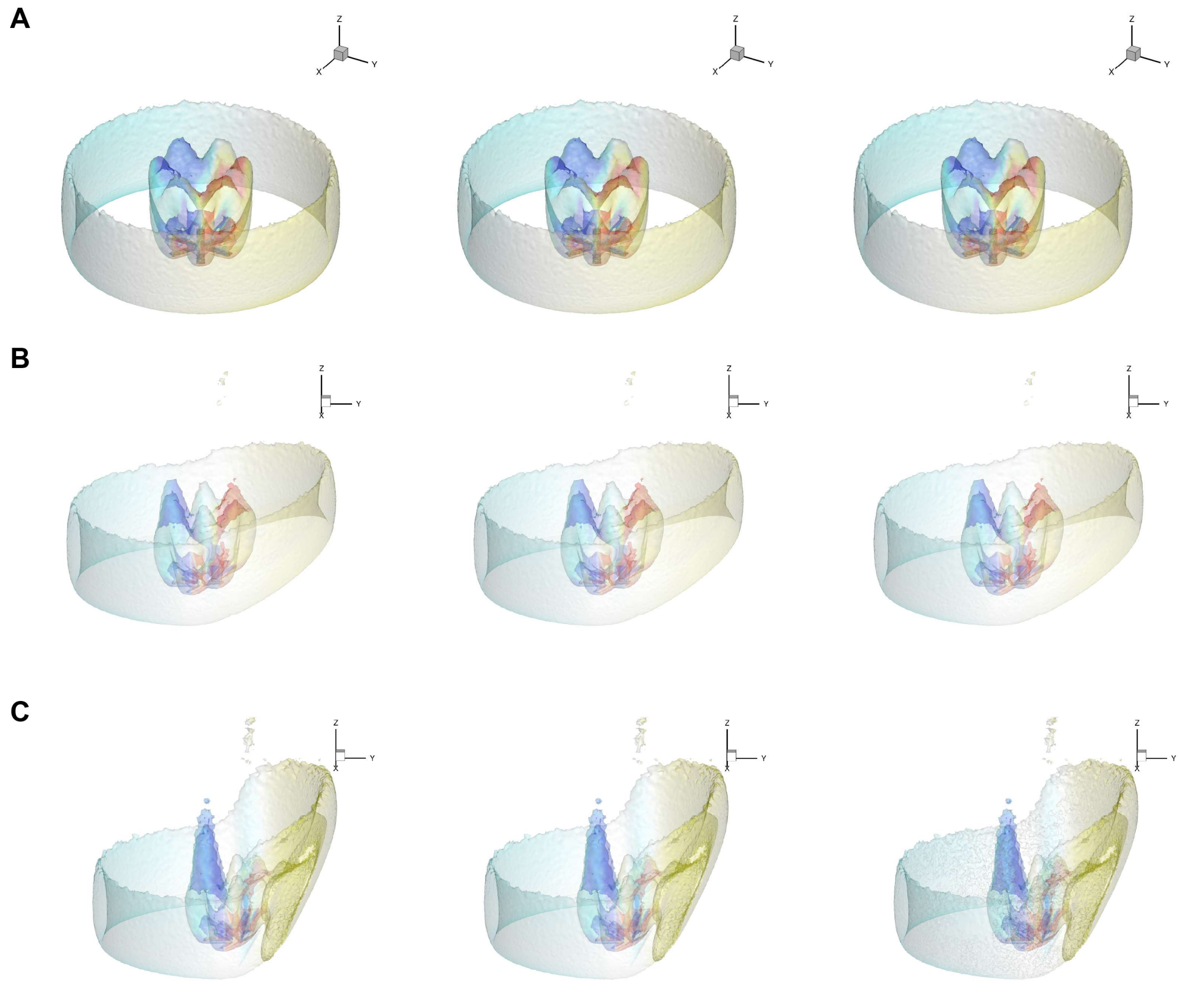

Fig. S23. 3D wake simulation. Consecutive snapshots of isosurfaces of Q-criterion (Qd^2/u_t^2) coloured by vorticity ($\omega_x d/t$), ranging from −5 (blue) to +5 (red), from high-fidelity CFD simulations exhibiting (**A**) a steady wake for symmetric dandidrone with opening angle of 180°, (**B**) a steady wake for asymmetric dandidrone with opening angle of 150°, and (**C**) a steady wake for asymmetric dandidrone with opening angle of 120°.

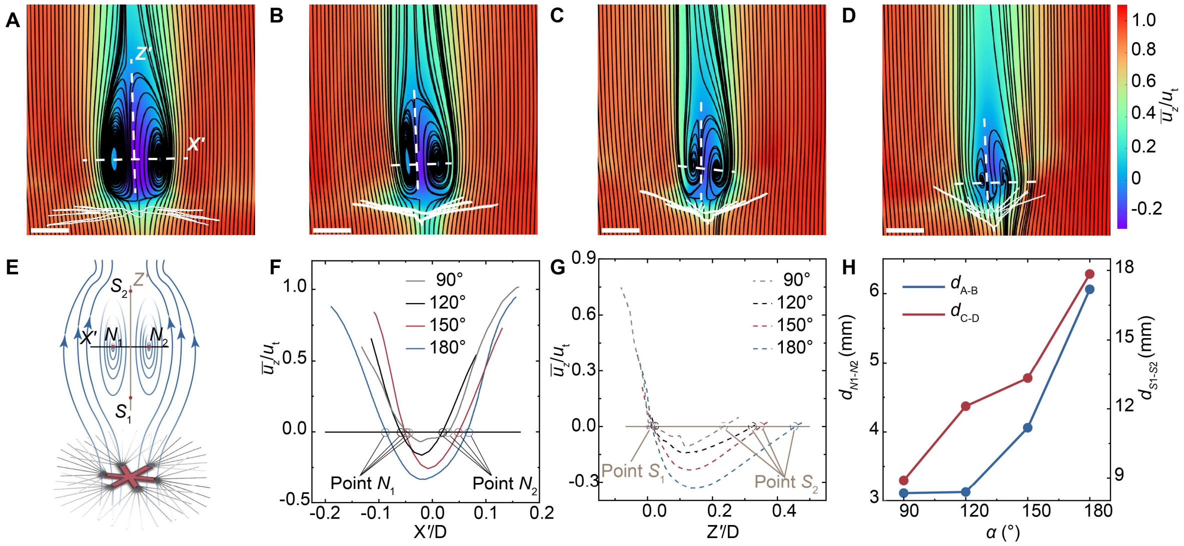

Fig. S24. Aerodynamic properties of the vortex ring of the dandidrone. Contours of time-averaged vertical velocity on the azimuthal plane of the dandidrone and streamlines for an opening angle of (**A**) 180°, (**B**) 150°, (**C**) 120°, and (**D**) 90°. (**E**) Topological points of the wake, including the left (N_1) and right (N_2) nodes, and the upstream (S_1) and downstream (S_2) saddle points. (**F**) Vertical velocity along the axis X through the nodes N_1 and N_2 for different values of the opening angle. (**G**) Vertical velocity along the axis Z through the saddle points S_1 and S_2 for different values of the opening angle. (**H**) Euclidean distance between the nodes N_1 and N_2, and between the saddle points S_1 and S_2 versus the opening angle. All the scale bars are 0.5 cm.

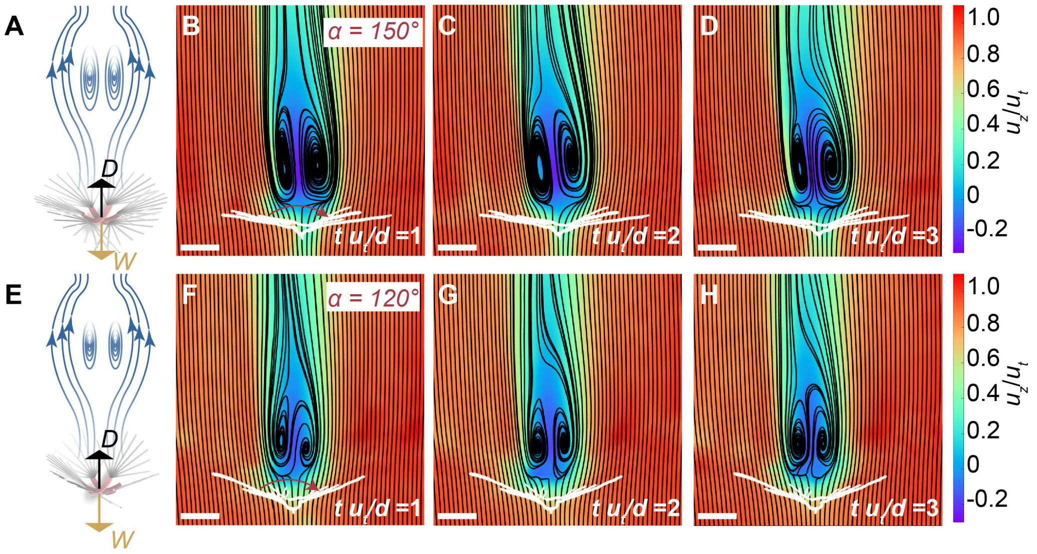

Fig. S25. The SVR stability at different opening angles. The schematic of the dandidrone with an opening angle of 150° (**A**). Contours of the instantaneous vertical velocity and streamlines at instants $tu_t/d = 1$ (**B**), $tu_t/d = 2$ (**C**), and $tu_t/d = 3$ (**D**). The schematic of the dandidrone with an opening angle of 120° (E). Contours of the instantaneous vertical velocity and streamlines at instants $tu_t/d = 1$ (**F**), $tu_t/d = 2$ (**G**), and $tu_t/d = 3$ (**H**). Scale bars are 0.5 cm.

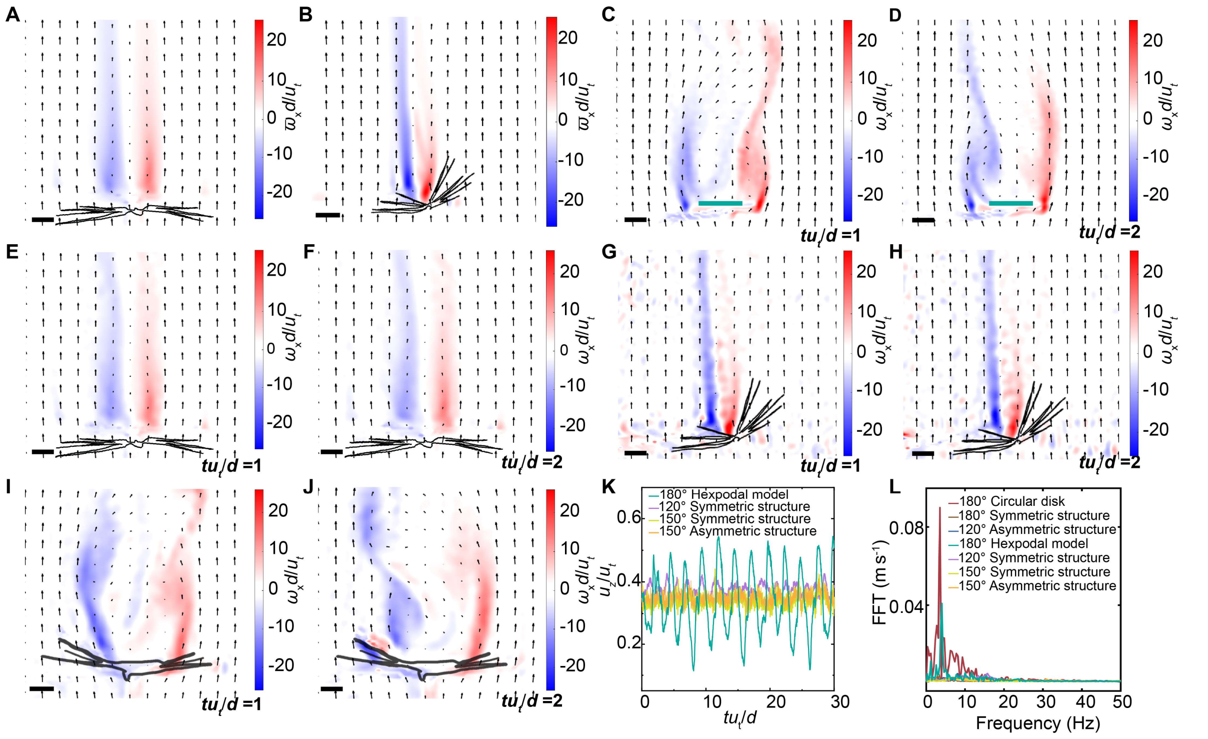

Fig. S26. Aerodynamic properties. (**A**) Contours of the time-averaged vorticity $\overline{\boldsymbol{\omega}}$ (nondimensionalised with u_t/d) around dandidrones with an opening angle of (**A**) 180° (fully open) and (**B**) 120° (asymmetric configuration). Contours of the instantaneous vorticity ω around the hexapodal core without filaments at two instants one convective period apart, namely (**C**) tu_t/d = 1 and (**D**) tu_t/d = 2. Contours of ω around a dandidrone with an opening angle of 180° at (**E**) tu_t/d = 1 and (**F**) tu_t/d = 2. Contours of ω around a dandidrone with a 120° asymmetric opening angle at (**G**) tu_t/d =1 and (**H**) tu_t/d = 2. Contours of ω around a dandidrone with an added circular disk at its centre at (**I**) tu_t/d = 1 and (**J**) tu_t/d = 2. (**K**) Time series of the vertical velocity in the wake of various dandidrones (see legend) measured one diameter downstream (Z/d = 1). (**L**) Fast Fourier transform of the vertical velocity measured in the wake of various dandidrones (Z/d = 1) versus the frequency. Scale bars are 0.5 cm.

Supplementary Note S3: Aerodynamic Property of Dandidrone

The drag coefficient (C_D) of the dandidrone was defined as

$$C_{\mathrm{D}} = \frac{W}{\frac{1}{2}\rho u_{\mathrm{t}}^2 A} \quad (1)$$

where the weight W was measured using an Ohaus Explorer analytical balance with a resolution of 0.1 mg. The air density is taken as ρ = 1.204 kg m^{-3}, corresponding to standard conditions at 20 °C and 1 atm; A is the projected area of the dandidrone, including both the projected area of the filamentous structure and of the central active component. The terminal velocity u_{t} was evaluated through drop tests (Materials and Methods). By adding weight to different models, it was possible to measure how the terminal velocity varied with the weight. This is shown in the fig. S27, where the Reynolds number is

$$Re = \frac{u_{\mathrm{t}} d}{\nu} \quad (2)$$

with d being the diameter of the circumscribed circle to the pappus, and $\nu = 15.11 \times 10^{-6}\ \mathrm{m^2\ s^{-1}}$ is the kinematic viscosity of the air at 20 °C and 1 atm.

To be able to observe the untethered flier in a fixed position with respect to a laboratory-fixed frame, a vertical wind tunnel was built (fig. S30). By setting the speed of the upward flow stream in the wind tunnel at the same value as the magnitude of the terminal velocity u_{t} , the dandidrone hovered at a constant position in the tunnel. The wind tunnel velocity was measured by particle image velocimetry.

The dandidrone was then exposed to different light intensities to change the opening angle (fig. S28). As the opening angle is increased or decreased, the dandidrone moves upwards or downwards, respectively, because the wind tunnel speed is no longer the same as the terminal velocity. By keeping the illumination constant, and thus the opening angle fixed, the terminal velocity for each opening angle could be measured by identifying the wind tunnel speed allowing the dandidrone to remain at a constant vertical position in the wind tunnel.

While hovering at a constant height, the dandidrone rotated around its axisymmetric axis at a modest angular velocity of 1.68 ± 1.0 ° s^{-1} (fig. S32). The rotation behaviour was recorded using a digital single-lens reflex camera (Canon EOS 60D) operating at 25 frames per second. Dandidrone trajectories were analysed using TRACKER software to determine angular velocity. The remarkable rotatory stability of the dandidrone is due to the precise axisymmetric structure. In contrast, its natural counterpart, the dandelion seed's diaspore, revealed an angular velocity of 50.8 ± 17.7 ° s^{-1}.

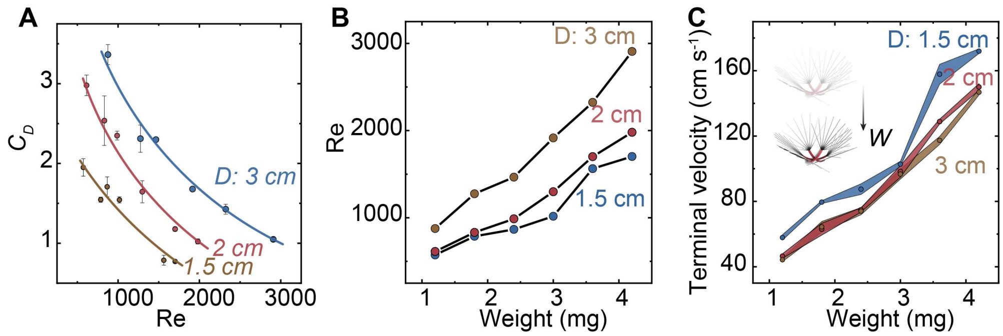

Fig. S27. The aerodynamics of dandidrones. (**A**) Drag coefficient of the dandidrone as a function of the Reynolds number. (**B**) Reynolds number and (**C**) terminal velocity of dandidrones with different diameters under different loads. Error bars are displayed as the mean value +/- one standard deviation (n = 3). The same sample was measured repeatedly.

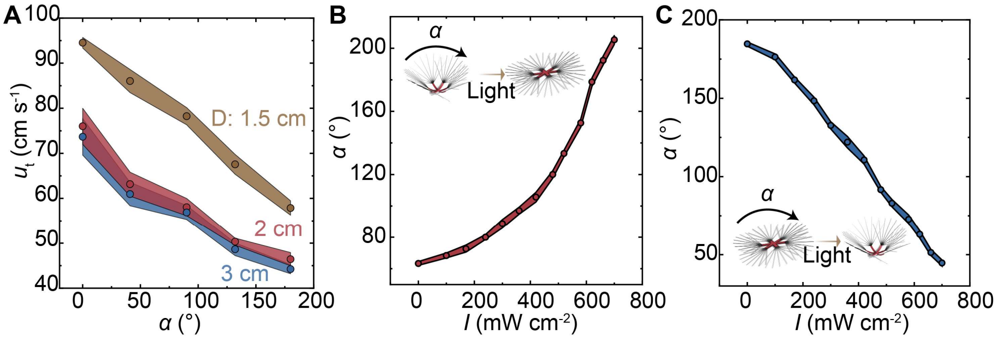

Fig. S28. Light-induced shape and terminal velocity changes of dandidrones. (**A**) The variation of terminal velocity at different opening angles for different sample sizes. The change in opening angle in the dandidrone with an initially (**B**) closed and (**C**) open configuration as a function of the light intensity. Error bars are displayed as the mean value +/- one standard deviation (n = 3). The same sample was measured repeatedly.

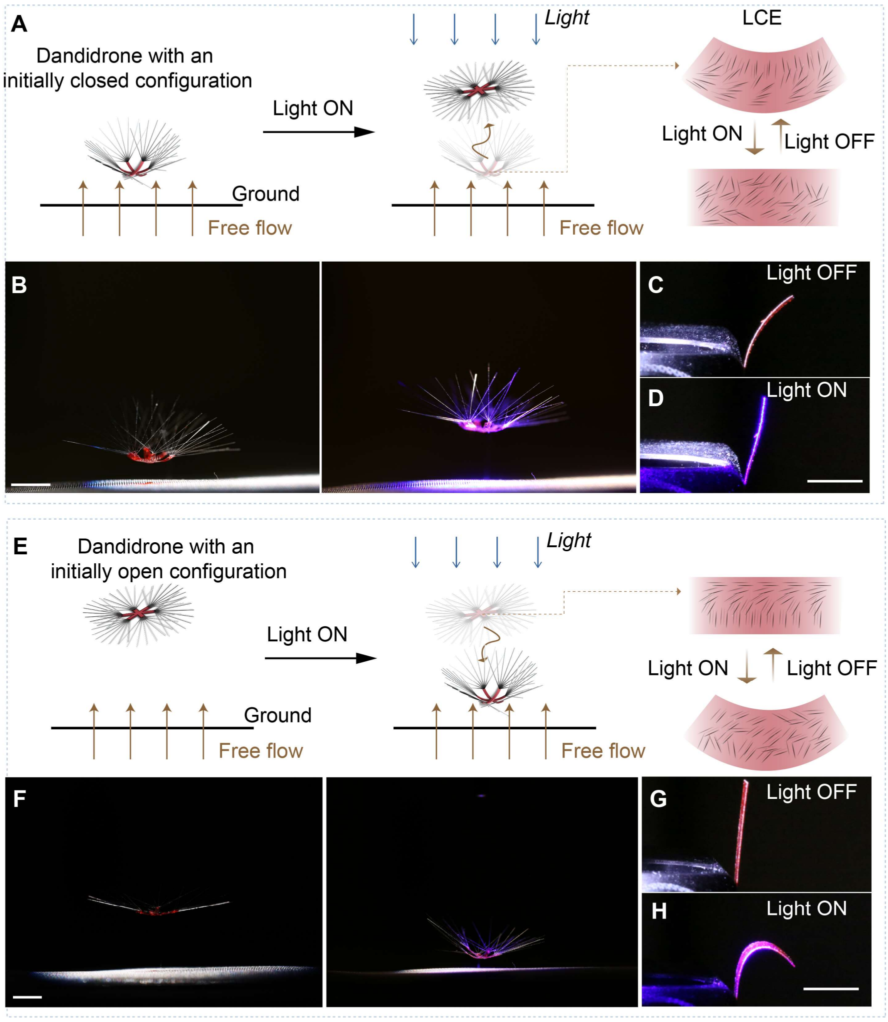

Fig. S29. The two distinct structural configurations of the dandidrone. (**A**) The schematic of the dandidrone with an initially closed configuration, increasing the height under the light illumination. The molecular orientation of corresponding LCE strip with an initially bending configuration is shown on the right. (**B**) Light-induced upward movement of the dandidrone with an initially closed configuration. The image is identical to that shown in Fig. 1D and is reused here to illustrate the light-induced structural opening and upward motion of the dandidrone. The photographs of (**C**) a bent LCE strip before light illumination and (**D**) a flattening strip upon light illumination. (**E**) The schematic of the dandidrone with an initially open configuration, decreasing in height under the light. The molecular orientation of the corresponding LCE strip is shown on the right side. (**F**) Light-induced downward movement of the dandidrone with an initially open configuration. The photographs of an LCE strip exhibiting (**G**) a flat shape before light illumination and (**H**) a bend strip upon light illumination. Wind tunnel speed: 0.6 m s^{-1}. The light intensity: 700 mW cm^{-2}. All the scale bars are 5 mm.

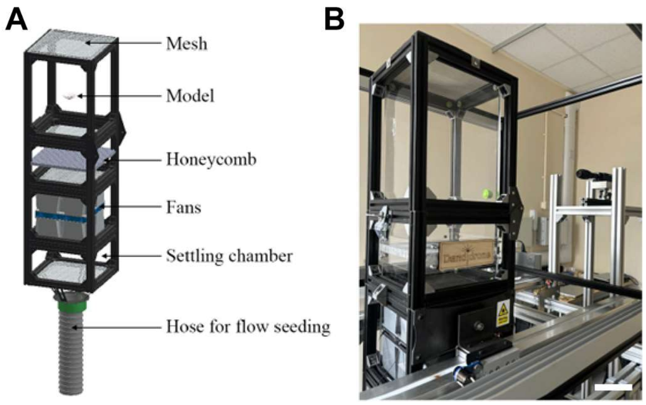

Fig. S30. The vertical wind tunnel. (**A**) Schematic drawings of the vertical wind tunnel. The model in the test section is not to scale. The air flows in the upward direction. (**B**) Photograph of experimental setup in laboratory.

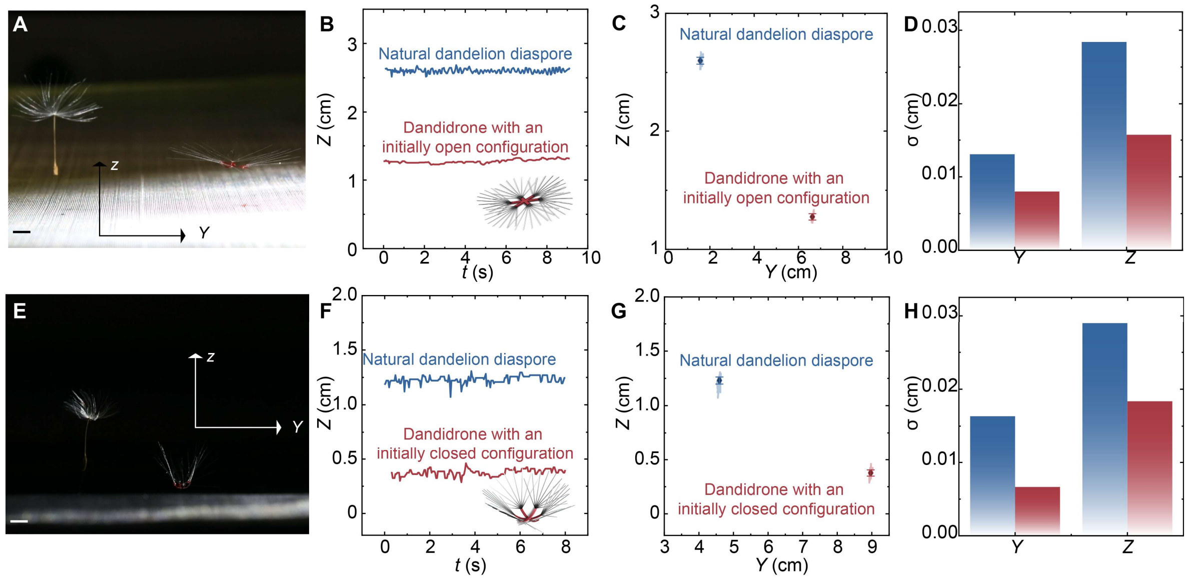

Fig. S31. The stability of the dandidrone. (**A**) Photographs of a natural dandelion diaspore (left) and a dandidrone (right) with an initially open configuration floating in the wind tunnel. (**B**) The vertical coordinate of the natural dandelion diaspore (blue) and the dandidrone with an initially open configuration (red) over time. (**C**) The *Y* and *Z* coordinates of the natural dandelion diaspore (blue) and the dandidrone with an initially open configuration (red) over 1 s (25 data points). (**D**) The standard deviation of the vertical and horizontal coordinates of the natural dandelion diaspore and the dandidrone with an initially open configuration. (**E**) Photographs of a natural dandelion diaspore (left) and a dandidrone (right) with an initially closed configuration floating in the wind tunnel. (**F**) The vertical coordinate of the natural dandelion diaspore (blue) and the dandidrone with an initially closed configuration (red) over time. (**G**) The *Y* and *Z* coordinates of the natural dandelion diaspore (blue) and the dandidrone with an initially closed configuration (red) over 1 s (25 data points). (**H**) The standard deviation of the vertical and horizontal coordinates of the natural dandelion diaspore and the dandidrone with an initially closed configuration. Wind tunnel speed: 0.6 m s^{-1}. All the scale bars are 5 mm.

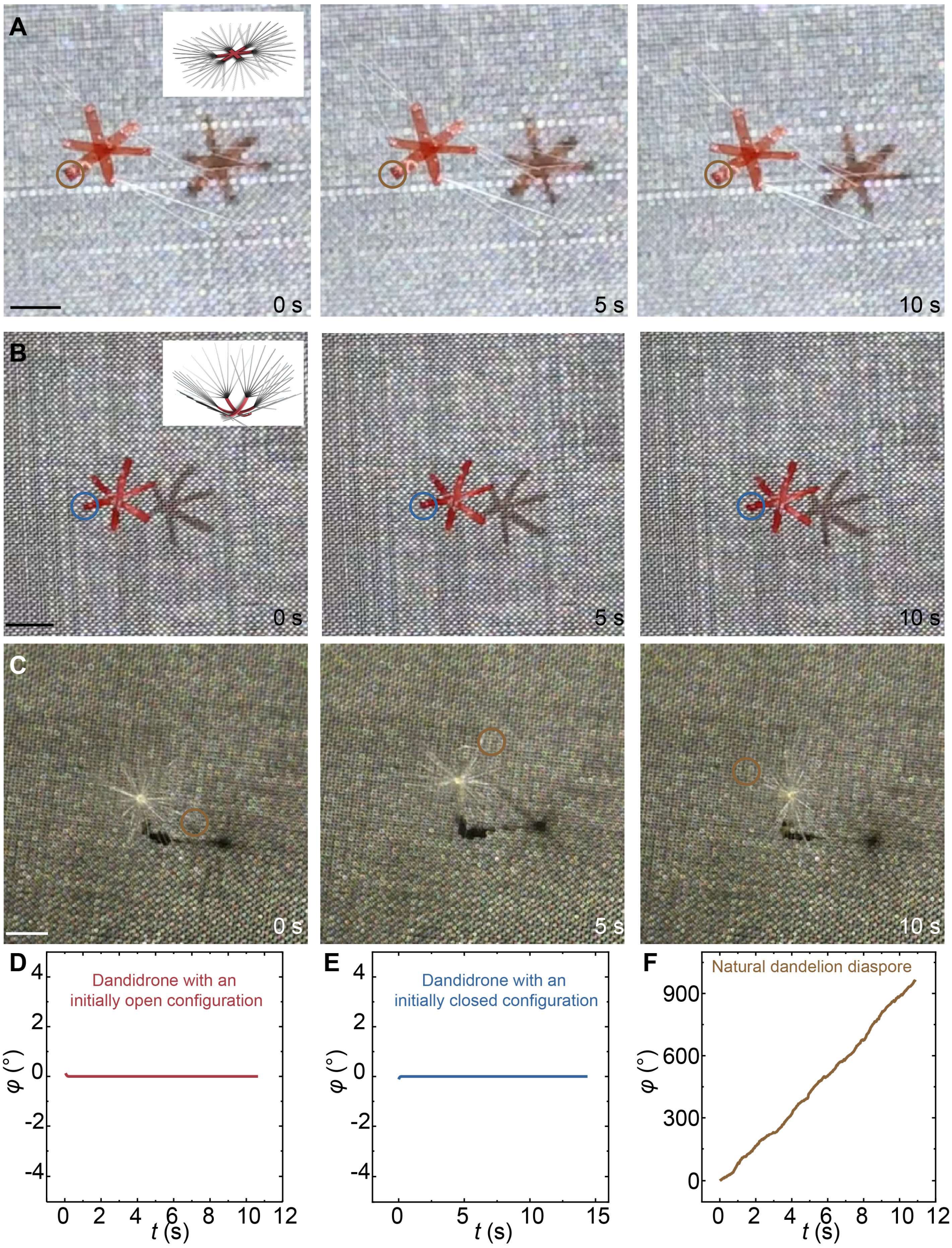

Fig. S32. The rotatory stability of dandidrones. Snapshots of the dandidrone with an initially (**A**) open and (**B**) closed configuration. (**C**) Snapshots of a natural dandelion diaspore. The change in rotation angle φ of the dandidrone with an initially (**D**) open and (**E**) closed configuration with time. (**F**) The change of φ of a natural dandelion diaspore with time. Wind tunnel speed: 0.6 m s^{-1}. Scale bar: 5 mm.

Supplementary Note S4: The Agile Manoeuvring of Dandidrone

Vertical Motion. Under uniform optical irradiation, the dandidrone undergoes symmetric deformation (fig. S33), resulting in a change in the aerodynamic drag that enables the vertical motion — either ascent or descent (figs. S34 and S35). The direction of motion is determined by the initial geometric configuration (fig. S29). Specifically, for the dandidrone with an initially open configuration, optical stimulation induces structural closure, leading to a descent motion. In contrast, when the structure begins in a closed state, uniform illumination prompts structural opening, resulting in an ascent motion.

Flipping. A uniform sustained illumination on an initially closed state induces progressive opening of the structure, ultimately driving the opening angle to exceed 180° and eventually triggering in-air flipping (figs. S44 and S45).

Target Height. One can keep a dandidrone at a constant height by providing illumination at that height (Fig. 3A-3D and figs. S42). For example, a dandidrone with an initially closed configuration above the illuminated height falls up to the illuminated height, where it opens and rises again. The dandidrone exhibits oscillatory behaviour around that altitude, which serves as a tuneable upper or lower bound depending on the initial configuration (figs. S41 and S43).

Horizontal Motion. In contrast, the selective illumination of different parts of the dandidrone induces asymmetric deformation (figs. S36 and S46), generating lateral propulsion along the horizontal plane (figs. S37 and S39). Horizontal flight is initiated when the light intensity exceeds a threshold upon onset (figs. S38 and S40), after which the motion is sustained and governed by the scanning speed of the light source (fig. S47), enabling controllable navigation, while maintaining high stability in orientation (non-spinning) and vertical position during flight (fig. S48).

Selective local irradiation of specific structural components enables the programming of arbitrary trajectories, such as the formation of letter-like patterns (*e.g.,* A, B, C), as shown in figs. S49 to S51.

Three-Dimensional Trajectories. Together, vertical and horizontal controllable motions allow for the programming of complex three-dimensional trajectories. For instance, a dandidrone can be elevated to a designated height, translated laterally, and then guided downward (figs. S58 and S59), showcasing both the precision and stability of the feedback-driven actuation strategy.

Clustering. When two or three structures are brought into proximity, the negative pressure generated above the dandidrone leads to mutual attraction, forming a cohesive unit that can be manoeuvered as a single entity (figs. S52 to S57).

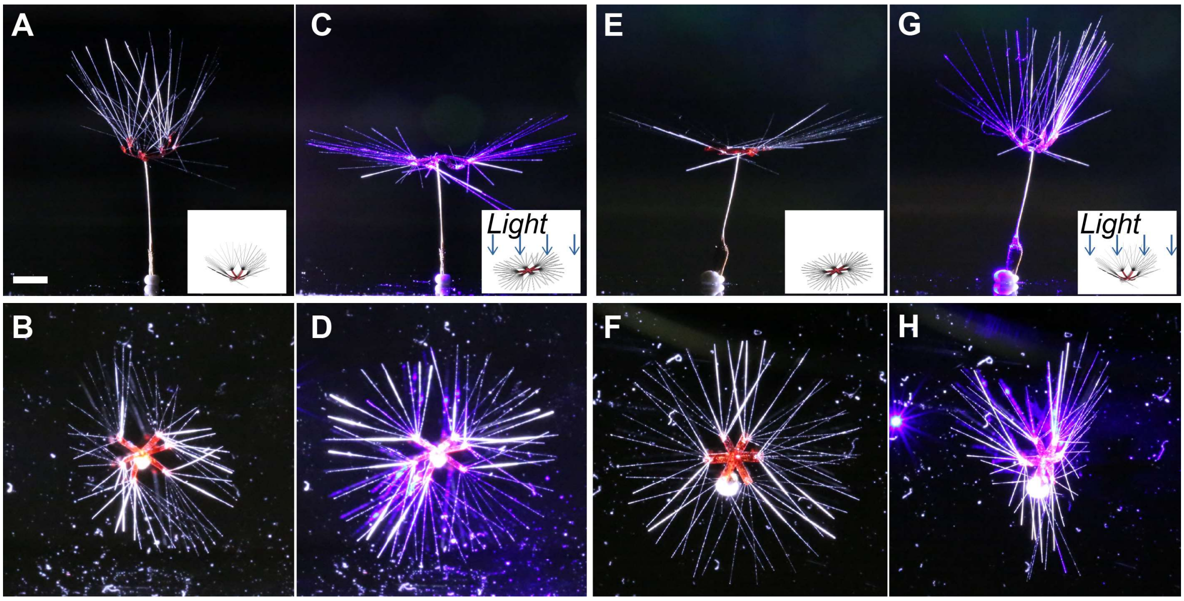

Fig. S33. The light-induced symmetric shape-morphing of dandidrones. (**A**) Side-view and (**B**) top-view photographs of the dandidrone with an initially closed configuration. The image in (**A**) is identical to that shown in fig. S17C and is reused here to represent the side-view of the dandidrone with an initially closed configuration (without light irradiation). (**C**) Side-view and (**D**) top-view photographs of the dandidrone with an initially closed configuration upon light irradiation. (**E**) Side-view and (**F**) top-view photographs of the dandidrone with an initially open configuration. The image of (**F**) is identical to that shown in fig. S17F and is reused here to maintain consistency in representing the top-view of the dandidrone with an initially open configuration. (**G**) Side-view and (**H**) top-view photographs of the dandidrone with an initially open configuration upon light irradiation. Light intensity: 600 mW cm^{-2}. Scale bars are 5 mm.

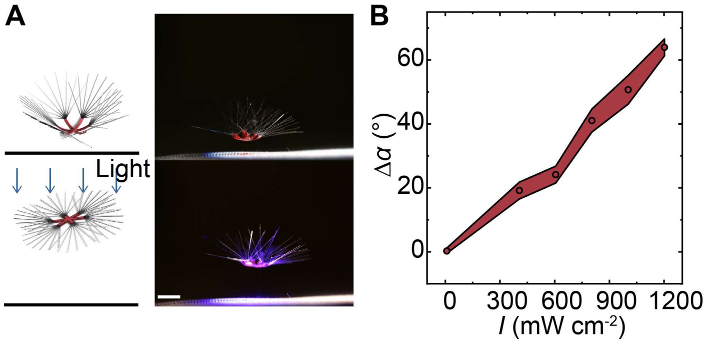

Fig. S34. The vertical displacement of an initially closed dandidrone. (**A**) Schematic drawing (left) and pictures (right) of a dandidrone with an initially closed configuration with light off (top) and on (bottom). The image is identical to that shown in Fig. 1D and is reused here to illustrate the light-induced structural opening and upward motion of the dandidrone. (**B**) The opening angle change of the dandidrone upon different light intensities. Wind tunnel speed: 0.6 m s^{-1}. Error bars are displayed as the mean value +/- one standard deviation (n = 3). The same sample was measured repeatedly.

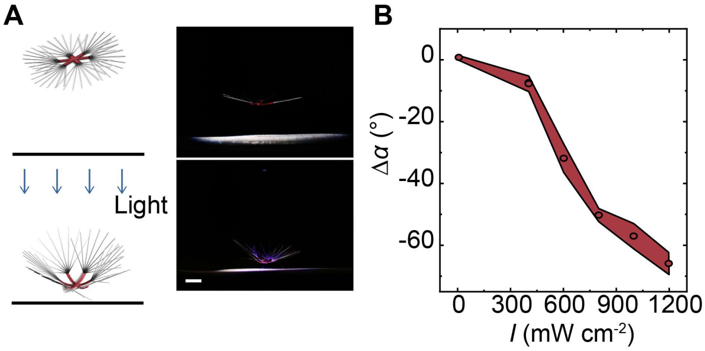

Fig. S35. The vertical displacement of an initially open dandidrone. (**A**) Schematic drawing (left) and pictures (right) of a dandidrone with an initially open configuration with light off (top) and on (bottom). The image is identical to that shown in fig. S29F and is reused here to illustrate the light-induced structural closing and downward motion of the dandidrone. (**B**) The opening angle of the dandidrone upon different light intensities. Wind tunnel speed: 0.6 m s^{-1}. Error bars are displayed as the mean value +/- one standard deviation (n = 3). The same sample was measured repeatedly.

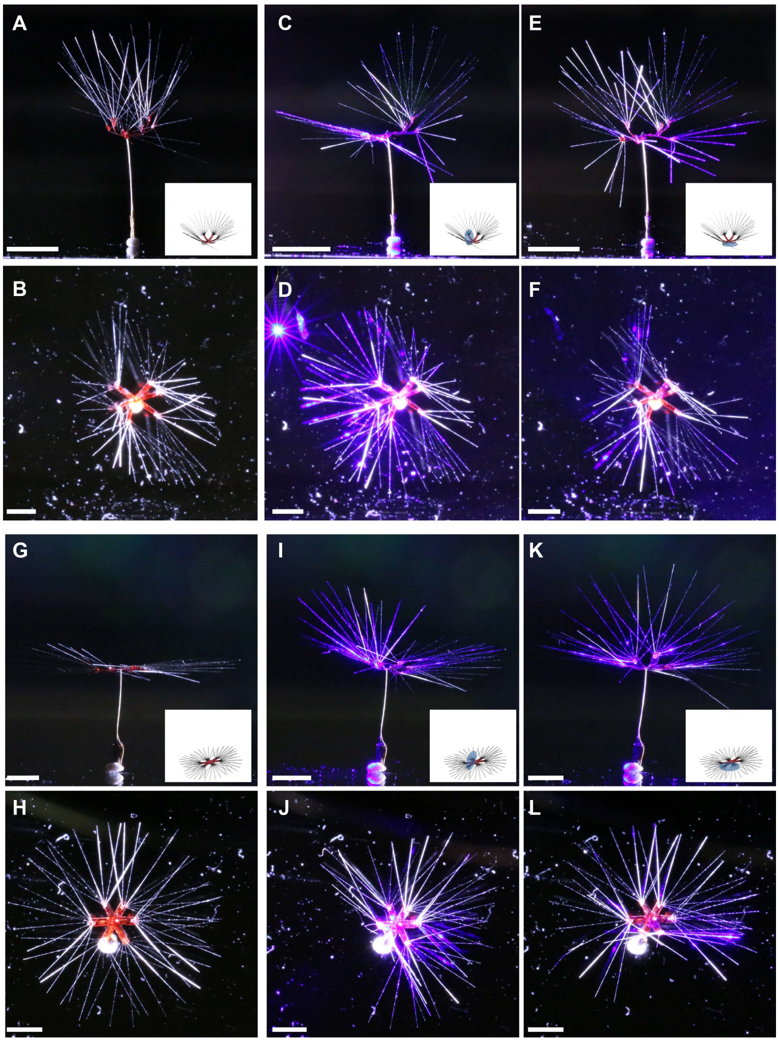

Fig. S36. The light-induced asymmetric shape-morphing of dandidrones. (**A**) Side-view and (**B**) top-view photographs of the dandidrone with an initially closed configuration. The image in (**A**) is identical to that shown in fig. S17C and is reused here to represent the side-view of the dandidrone with an initially closed configuration (without light irradiation). The image in (**B**) is identical to that shown in fig. S33B and is reused here to represent the top-view of the dandidrone with an initially closed configuration (without light irradiation). (**C**) Side-view and (**D**) top-view photographs of the dandidrone with an initially closed configuration upon light irradiation on left-side segments. (**E**) Side-view and (**F**) top-view photographs of the dandidrone with an initially closed configuration upon light irradiation on front segments. (**G**) Side-view and (**H**) top-view photographs of the dandidrone with an initially open configuration. The image in (**H**) is identical to that shown in fig. S17F and is reused here to maintain consistency in representing the top-view of the dandidrone with an initially open configuration. (**I**) Side-view and (**J**) top-view photographs of the dandidrone with an initially open configuration upon light irradiation on left-side segments. (**K**) Side-view and (**L**) top-view photographs of the dandidrone with an initially open configuration upon light irradiation on front segments. Light intensity: 600 mW cm^{-2}. Scale bars are 5 mm.

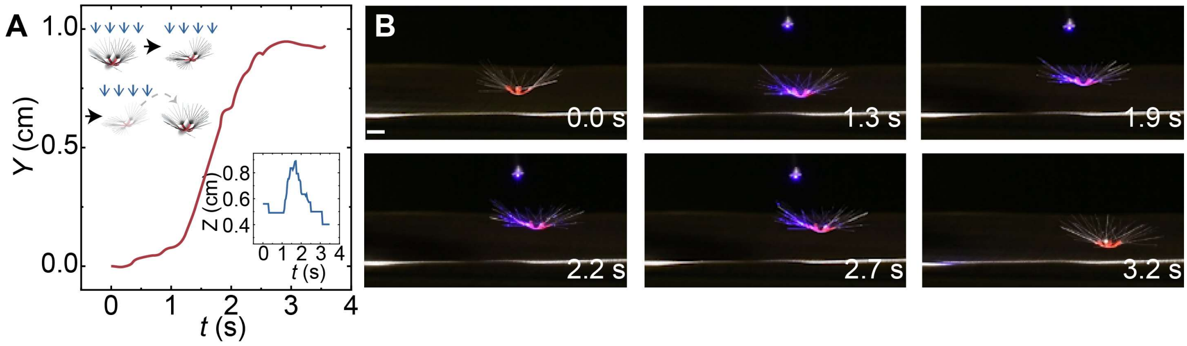

Fig. S37. The horizontal displacement of an initially closed dandidrone. (**A**) Time-history of the *Y* coordinate (and *Z* coordinate in the inset) of the dandidrone under light illumination. (**B**) Snapshot images of the dandidrone under light illumination. Wind tunnel speed: 0.6 m s^{-1}. Light intensity: 600 mW cm^{-2}. The scale bar is 5 mm.

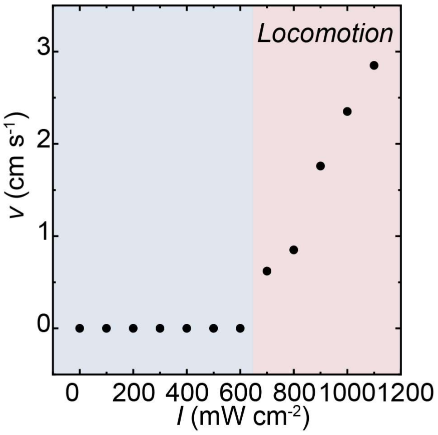

Fig. S38. The horizontal speed of an initially closed dandidrone. The horizontal speed of the dandidrone under different light intensities. Wind tunnel speed: 0.6 m s^{-1}.

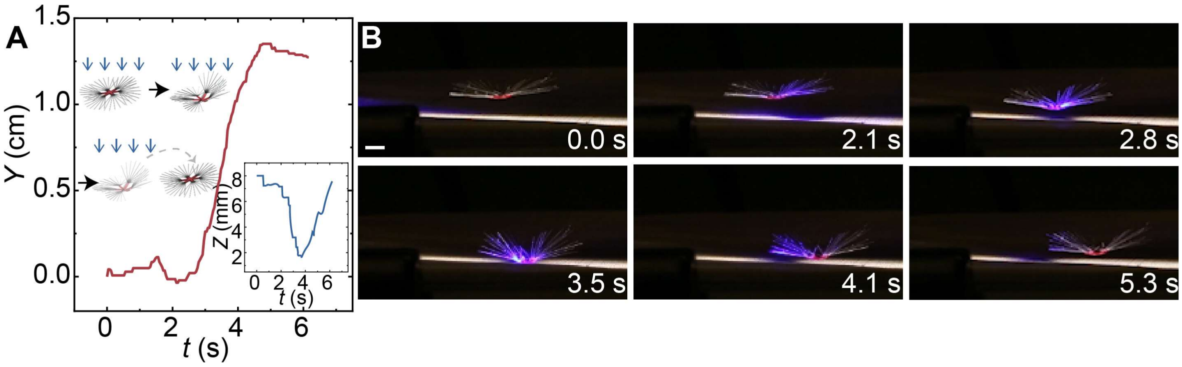

Fig. S39. The horizontal displacement of an initially open dandidrone. (**A**) Time-history of the *Y* coordinate (and *Z* coordinate in the inset) of the dandidrone under light illumination. (**B**) Snapshot images of the dandidrone under light illumination. Wind tunnel speed: 0.6 m s^{-1}. Light intensity: 600 mW cm^{-2}. The scale bar is 5 mm.

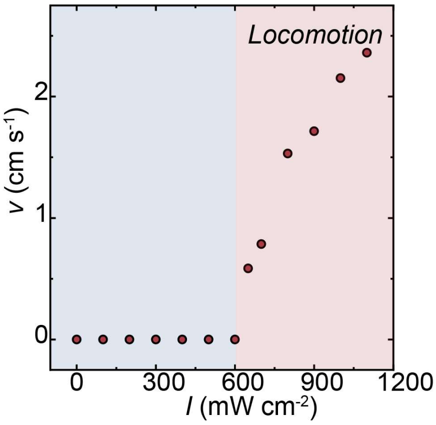

Fig. S40. The horizontal speed of an initially open dandidrone. The horizontal speed of the dandidrone under different light intensities. Wind tunnel speed: 0.6 m s^{-1}.

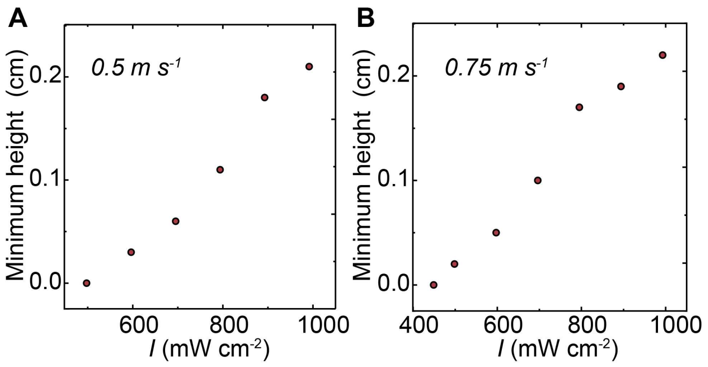

Fig. S41. The change of low height limit of an initially closed dandidrone. The minimum height of the dandidrone for different light intensities in a wind tunnel speed of 0.75 m s^{-1} for the same experiment as in Fig. 3A-3C.

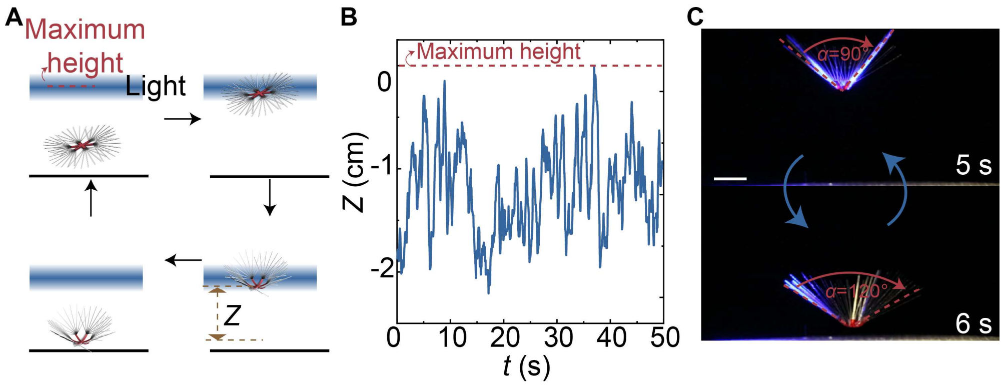

Fig. S42. Altitude self-regulation of an initially open dandidrone. (**A**) Schematic drawing of dandidrone with an optically set high-altitude limit. (**B**) Time history of the Z coordinate from the axis of the laser beam. (**C**) Snapshot images of the dandidrone's self-regulating opening angle while oscillating around the height limit. Terminal velocity without illumination: 0.6 m s^{-1}. Wind tunnel speed: 0.75 m s^{-1}. Light intensity: 650 mW cm^{-2}. Scale bar is 5 mm.

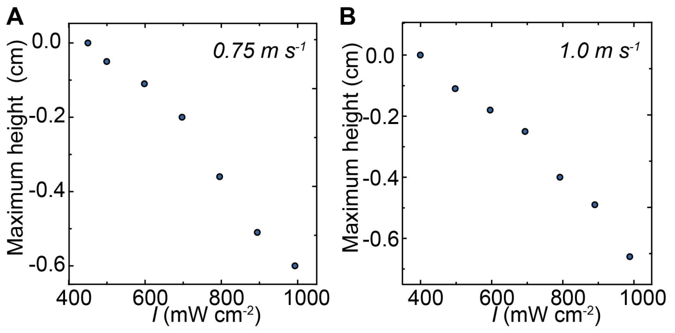

Fig. S43. The change of high height limit of the initially open dandidrone. The maximum height of the dandidrone for different light intensities in a wind tunnel speed of (**A**) 0.75 m s^{-1}, (**B**) 1.0 m s^{-1} for the same experiment as in fig. S42.

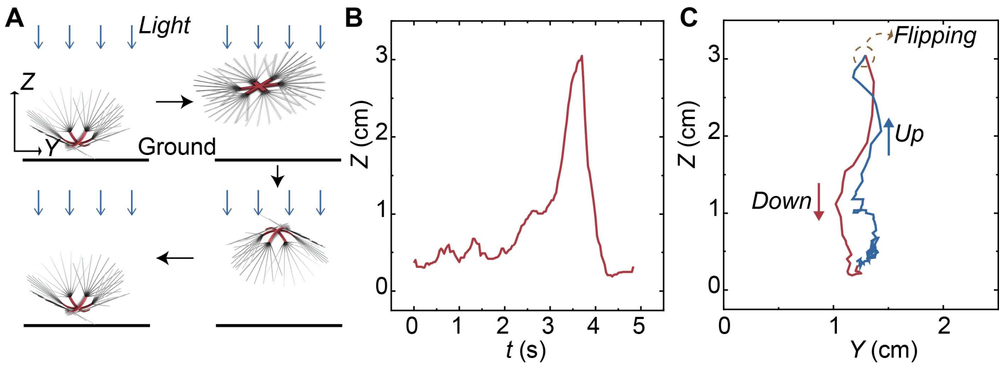

Fig. S44. The one-time flipping of a dandidrone. (**A**) The schematic drawing of a dandidrone flipping in the air. (**B**) The time history of the Z coordinate of the dandidrone during the flipping process. (**C**) The trajectory of the dandidrone flipping in the *Y*-*Z* plane.

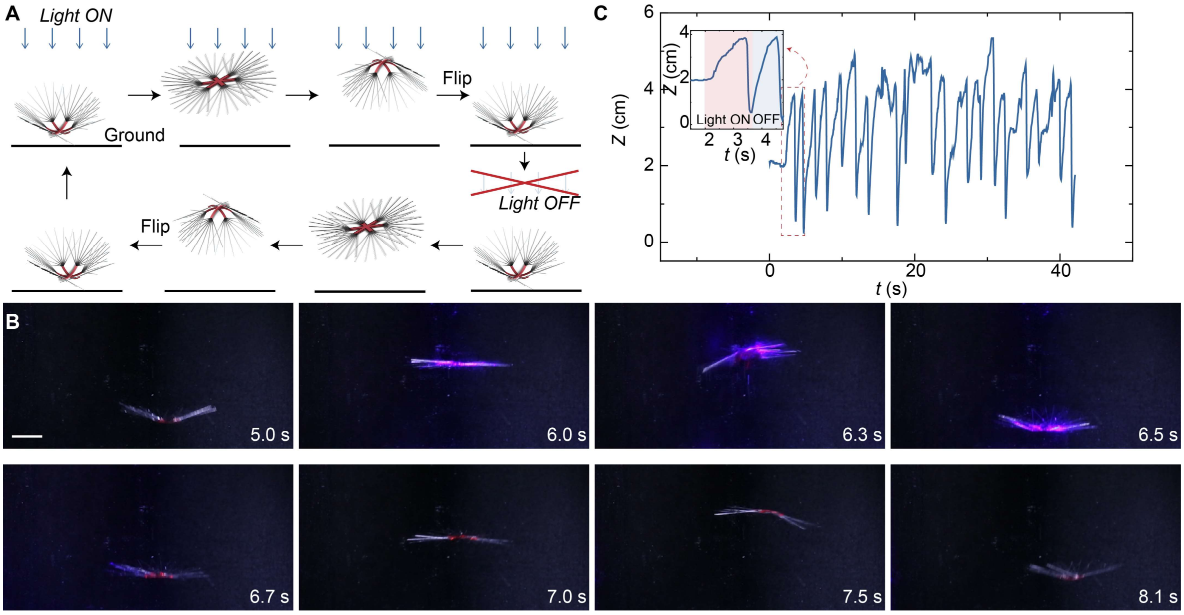

Fig. S45. The cyclic body flipping movement of dandidrone. (**A**) The schematic drawing of a dandidrone flipping upon light illumination and returning to its original position in the dark. (**B**) Pictures at different times of one cycle of flipping motion driven by light. (**C**) The *Z* coordinate of the dandidrone over more than 40 seconds, including 11 sequential flipping motions. Inset: the zoomed-in view of the dandidrone's Z coordinate during one flipping cycle. Wind tunnel speed: 0.7 m s^{-1}. Light intensity: 800 mW cm^{-2}. Scale bar is 5 mm.

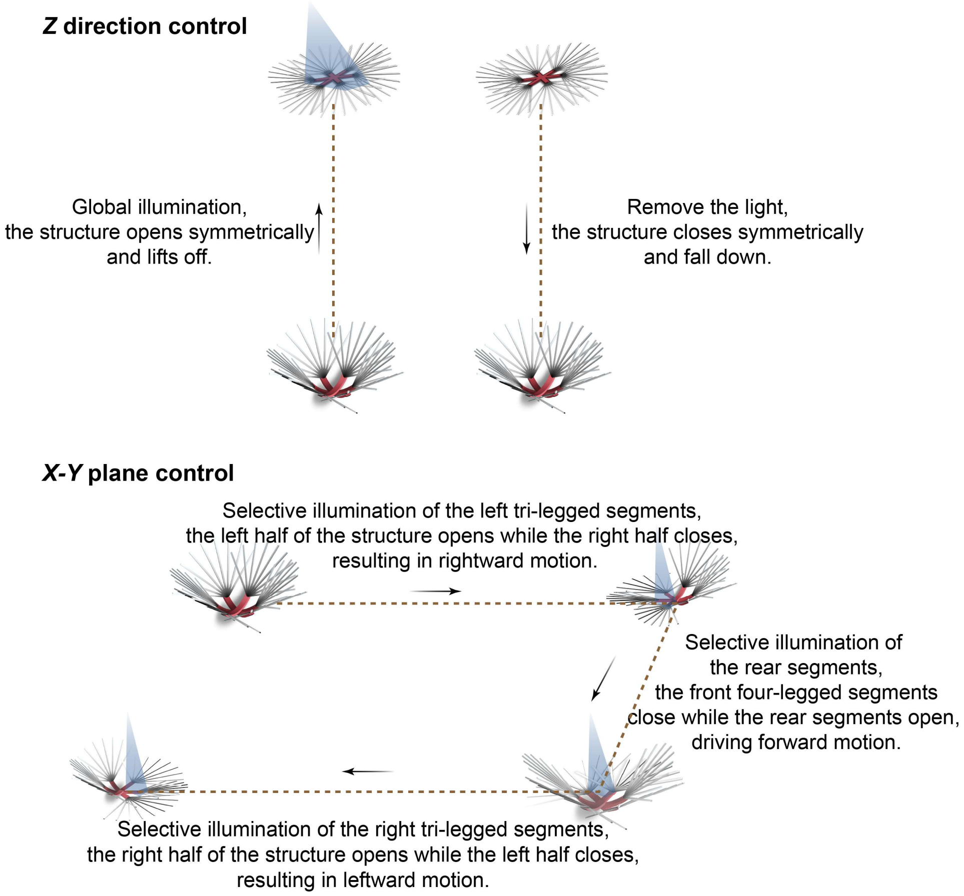

Fig. S46. Schematic of the *Z* direction (top) and *X-Y* plane (bottom) trajectory of the dandidrone and the corresponding light irradiation and control scheme.

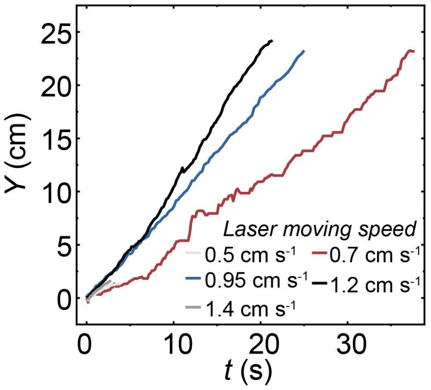

Fig. S47. Trajectories of the dandidrone over time at different laser scanning speeds.

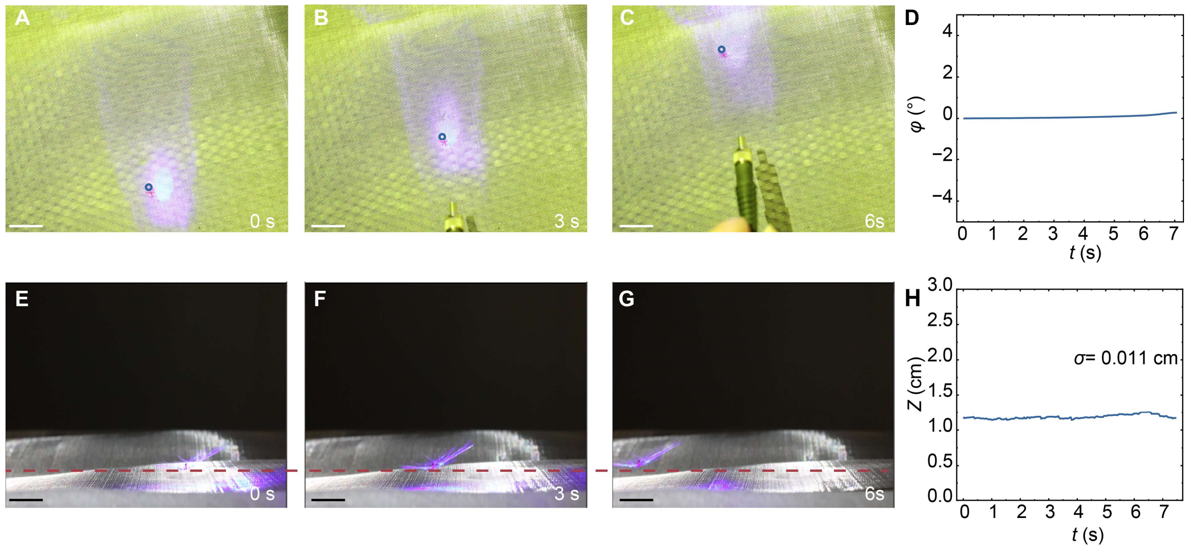

Fig. S48. The stability of dandidrones with asymmetric structure. (**A-C**) Snapshots of an asymmetrically configured dandidrone undergoing lateral motion under light illumination. (**D**) Time evolution of the rotation angle φ of the asymmetric dandidrone during light-induced motion. All the scale bars in (**A-C**) are 2 cm. (**E-G**) Snapshot images of the dandidrone under light illumination. (**H**) Time-history of the Z coordinate of the dandidrone under light illumination. All the scale bars in (**E-G**) are 1 cm. Wind tunnel speed: 0.6 m s^{-1}. Stability (σ) is standard deviation of vertical (Z) position over a 1 s time.

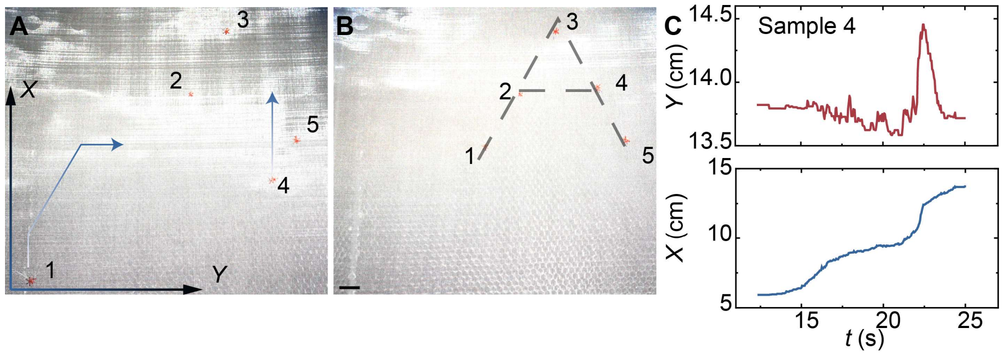

Fig. S49. The horizontal locomotion of dandidrones to form the letter A. (**A, B**) Photographs of the dandidrone moving in the *X*-*Y* plane. (**C**) The time histories of the *Y* (top) and *X* (bottom) coordinates of sample 4. Wind tunnel speed: 0.6 m s^{-1}. Light intensity: 700 mW cm^{-2}. Scale bar: 3 cm.

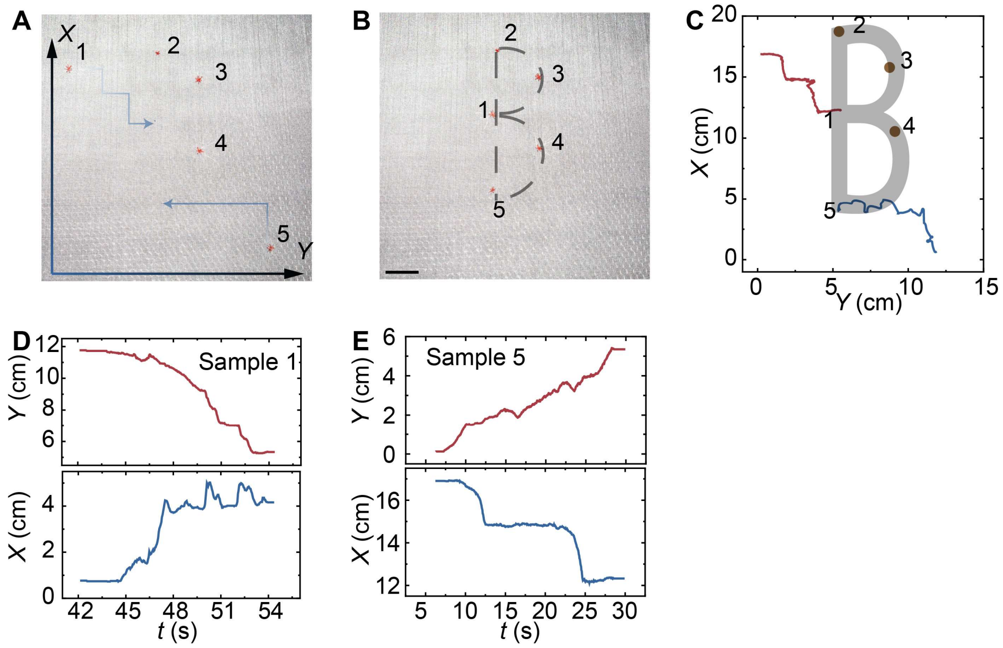

Fig. S50. The horizontal locomotion of dandidrones to form the letter B. (**A, B**) Photographs of the dandidrone moving in the *X*-*Y* plane. (**C**) The trajectory of two dandidrones steered to form the "B" shape. (**D**) The time histories of the *Y* (top) and *X* (bottom) coordinates of sample 1. (**E**) The time histories of the *Y* (top) and *X* (bottom) coordinates of sample 5. Wind tunnel speed: 0.6 m s^{-1}. Light intensity: 700 mW cm^{-2}. Scale bar: 3 cm.

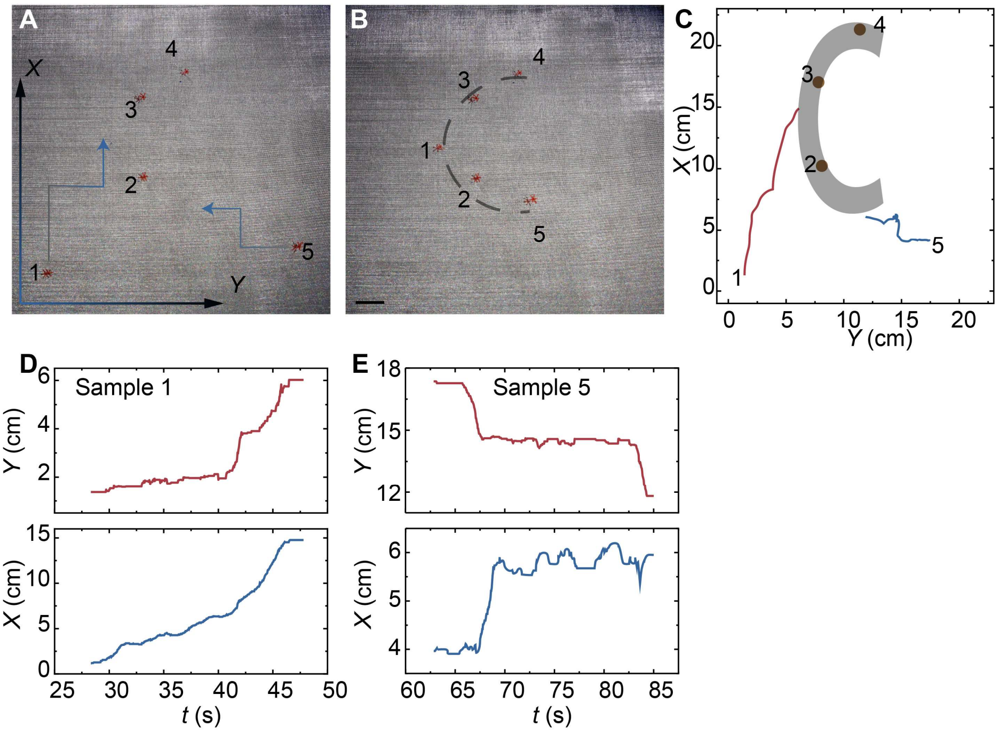

Fig. S51. The horizontal locomotion of dandidrones to form the letter C. (**A, B**) Photographs of the dandidrone moving in the *X*-*Y* plane. (**C**) The trajectory of two dandidrones steered to form the "C" shape. (**D**) The time histories of the *Y* (top) and *X* (bottom) coordinates of sample 1. (**E**) The time histories of the *Y* (top) and *X* (bottom) coordinates of sample 5. Wind tunnel speed: 0.6 m s^{-1}. Light intensity: 700 mW cm^{-2}. Scale bar: 3 cm.

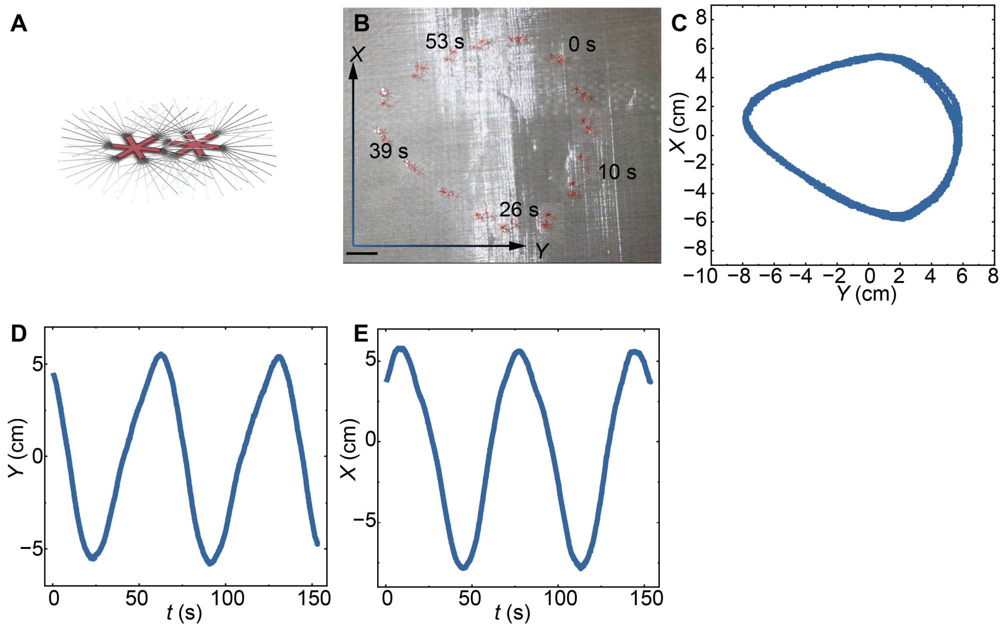

Fig. S52. The rotatory stability of the clustering of two dandidrones. (**A**) Schematic illustration of a two-dandidrone cluster. (**B**) Experimental image showing the orbital motion of the two-dandidrone cluster. (**C**) Corresponding orbital trajectory of the two-dandidrone cluster projected onto the *XY* plane. The time histories of the (**D**) *Y* and (**E**) *X* coordinates of the two-dandidrone cluster. Wind tunnel speed: 0.6 m s^{-1}. Scale bar: 2 mm.

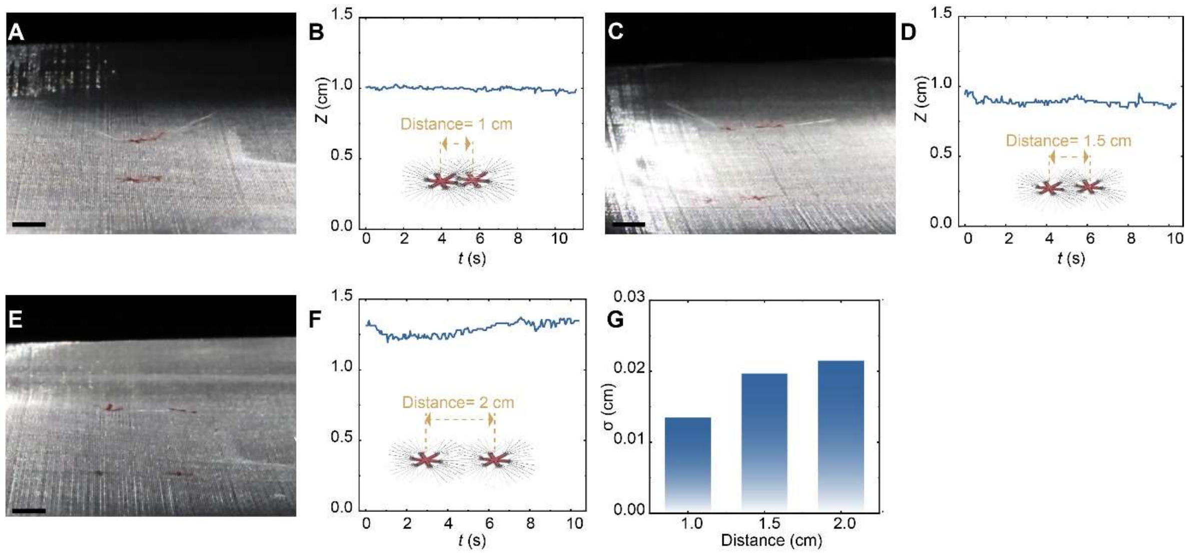

Fig. S53. The stability of the clustering of two dandidrones. (**A**) Photographs of a two-dandidrone cluster with a centre-to-centre distance of 1 cm. (**B**) The vertical coordinate of the two-dandidrone cluster with a centre-to-centre distance of 1 cm over time. (**C**) Photographs of a two-dandidrone cluster with a centre-to-centre distance of 1.5 cm. (**D**) The vertical coordinate of the two-dandidrone cluster with a centre-to-centre distance of 1.5 cm over time. (**E**) Photographs of a two-dandidrone cluster with a centre-to-centre distance of 2 cm. (**F**) The vertical coordinate of the two-dandidrone cluster with a centre-to-centre distance of 2 cm over time. (**G**) The standard deviation of the vertical coordinates of two-dandidrone cluster with different centre-to-centre distances. Wind tunnel speed: 0.6 m s^{-1}. All the scale bars are 5 mm.

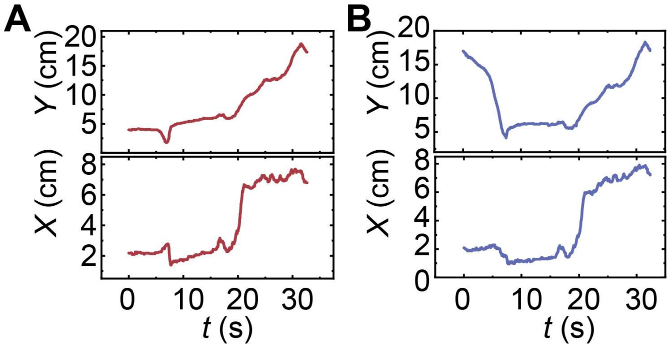

Fig. S54. The clustering of two dandidrones. (**A**) The time history of the *Y* (top) and *X* (bottom) coordinates of sample 1. (**B**) The time history of the *Y* (top) and *X* (bottom) coordinates of sample 2.

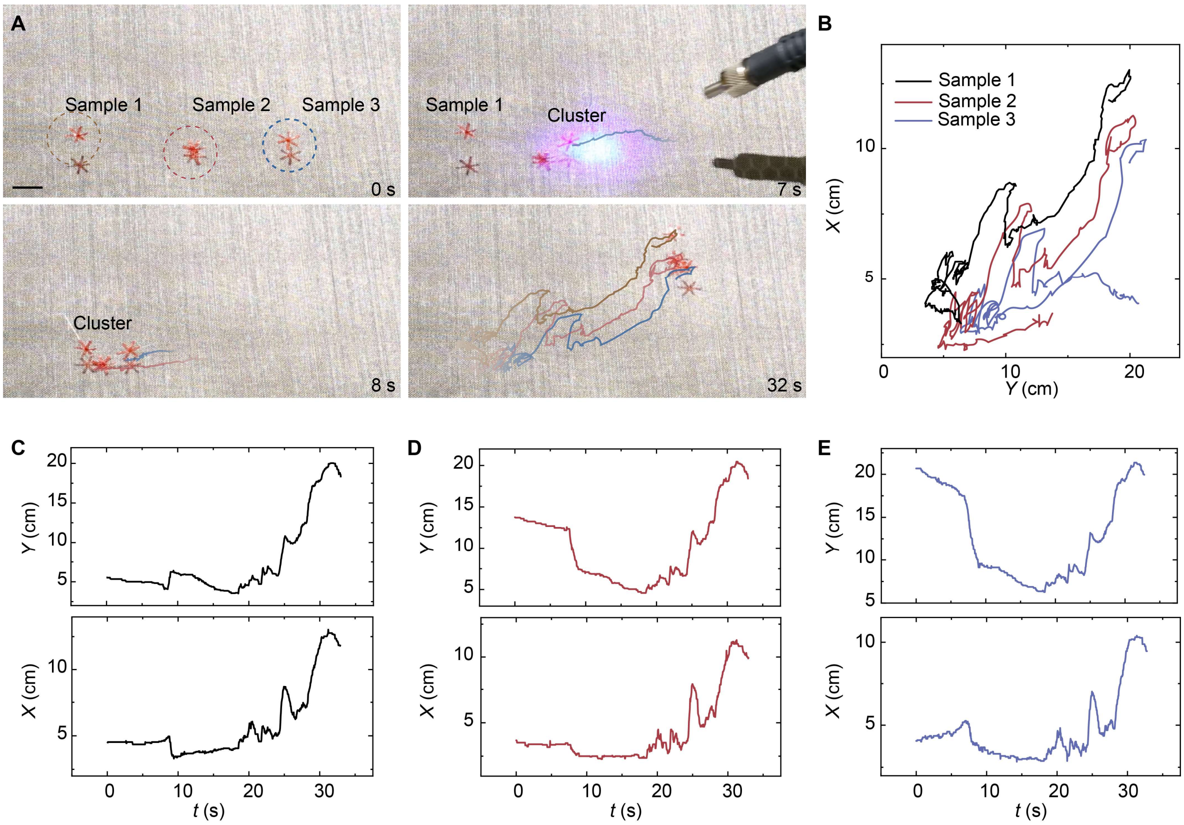

Fig. S55. Dandidrones swarms. (**A**) Snapshots of the clustering process among three dandidrones. (**B**) The trajectory of three dandidrones in the *X*-*Y* plane. The *Y* (top) and *X* (down) coordinates over time for sample 1 (**C**), sample 2 (**D**), and sample 3 (**E**). Wind tunnel speed: 0.6 m s^{-1}. Light intensity: 700 mW cm^{-2}. The scale bar: 0.5 cm.

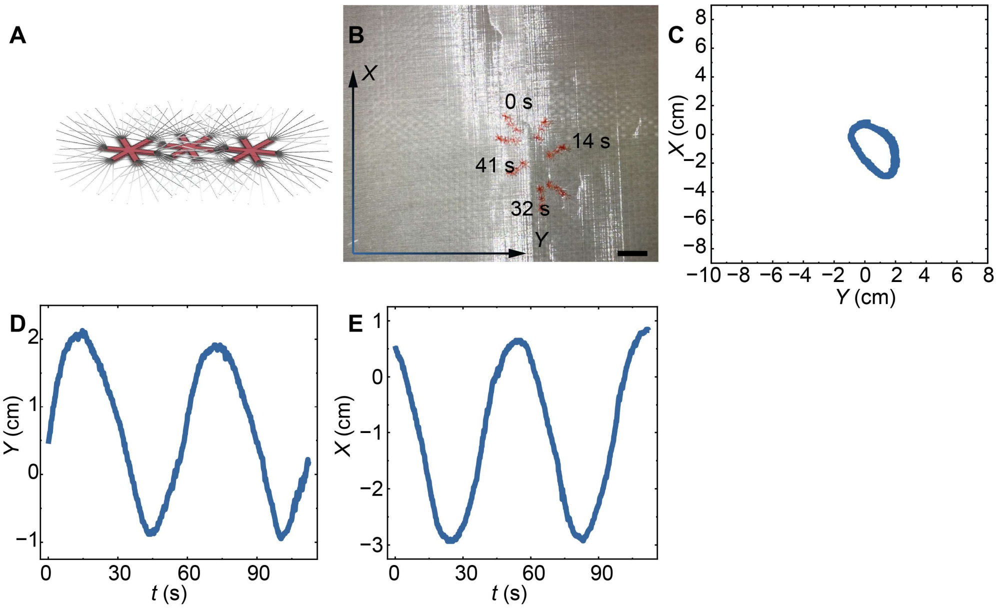

Fig. S56. The rotatory stability of the clustering of three dandidrones. (**A**) Schematic illustration of a three-dandidrone cluster. (**B**) Experimental image showing the orbital motion of the three-dandidrone cluster. (**C**) Corresponding orbital trajectory of the three-dandidrone cluster projected onto the *XY* plane. The time histories of the (**D**) *Y* and (**E**) *X* coordinates of the three-dandidrone cluster. Wind tunnel speed: 0.6 m s^{-1}. Scale bar: 2 mm.

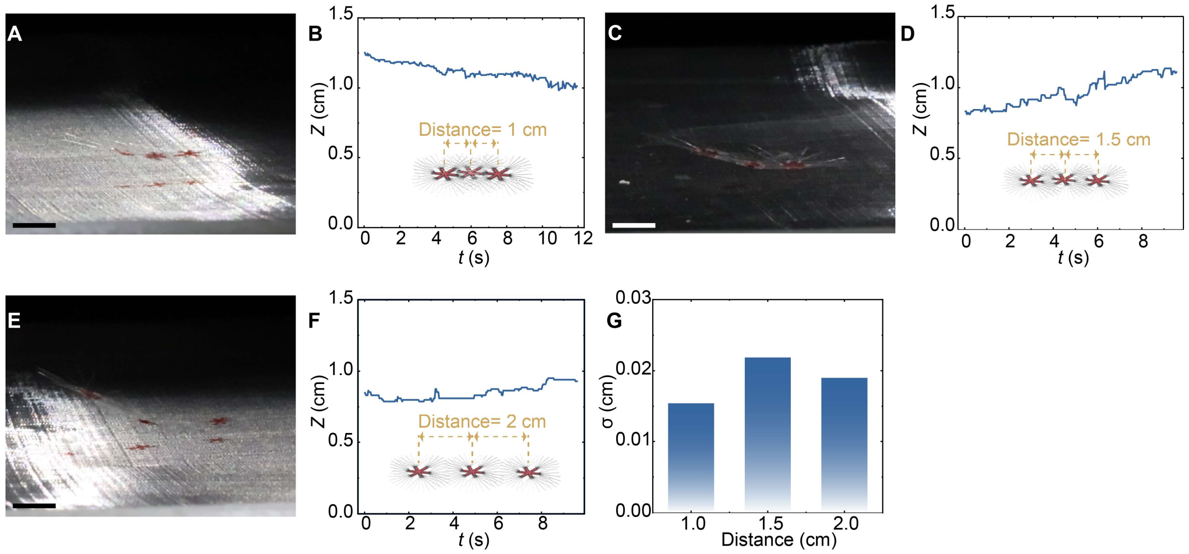

Fig. S57. The stability of the clustering of two dandidrones. (**A**) Photographs of a three-dandidrone cluster with a centre-to-centre distance of 1 cm. (**B**) The vertical coordinate of the three-dandidrone cluster with a centre-to-centre distance of 1 cm over time. (**C**) Photographs of a three-dandidrone cluster with a centre-to-centre distance of 1.5 cm. (**D**) The vertical coordinate of the three-dandidrone cluster with a centre-to-centre distance of 1.5 cm over time. (**E**) Photographs of a three-dandidrone cluster with a centre-to-centre distance of 2 cm. (**F**) The vertical coordinate of the three-dandidrone cluster with a centre-to-centre distance of 2 cm over time. (**G**) The standard deviation of the vertical coordinates of three-dandidrone cluster with different centre-to-centre distances. Wind tunnel speed: 0.6 m s^{-1}. All the scale bars are 5 mm.

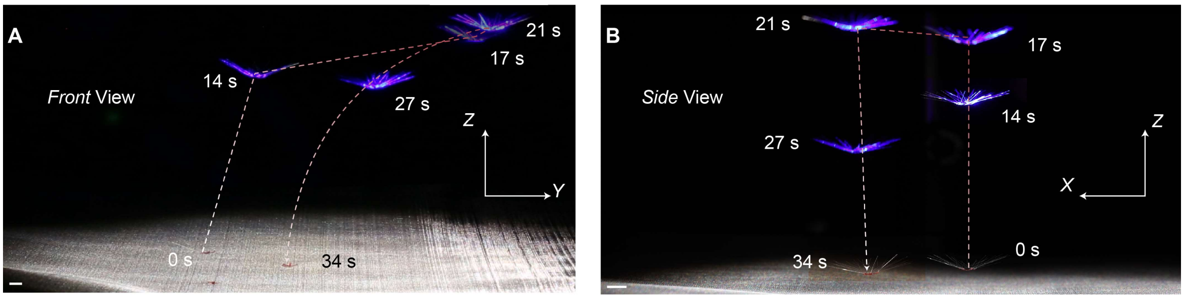

Fig. S58. The 3D locomotion of dandidrone. Snapshots of three-dimensional of (**A**) front view and (**B**) side view of locomotion of dandidrone driven by light. Wind tunnel speed: 0.6 m s^{-1}. Light intensity: 600 mW cm^{-2}. Scale bars are 5 mm.

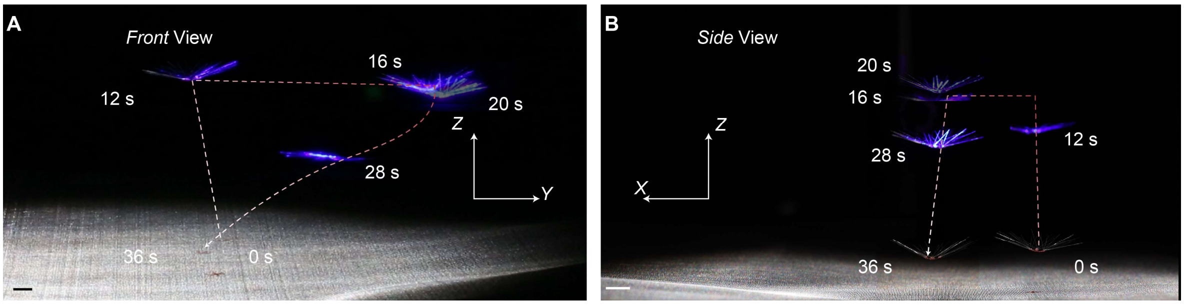

Fig. S59. The 3D locomotion of a dandidrone. Snapshots of dandidrones' locomotion through a three-dimensional trajectory; (**A**) front view and (**B**) side view. Wind tunnel speed: 0.6 m s^{-1}. Light intensity: 650 mW cm^{-2}. Scale bars are 5 mm**.**

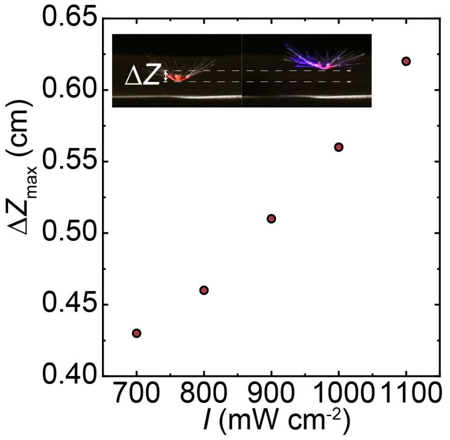

Fig. S60. The change in height of an initially closed dandidrone that is steered to move horizontally by selective illumination of one of its sides. The change in the Z coordinate of the dandidrone upon the different light intensities. The image (inset) is identical to that shown in fig. S37B and is reused here to highlight a different aspect of the motion (lateral versus longitudinal) arising from light-induced asymmetric deformation. Wind tunnel speed: 0.6 m s^{-1}.

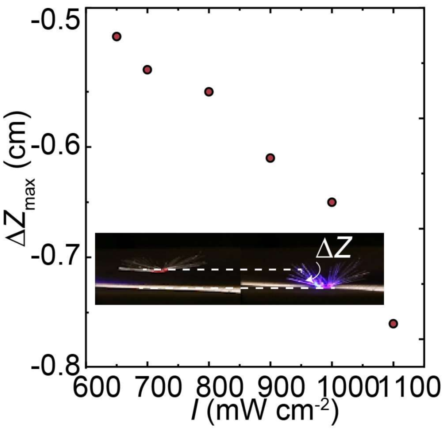

Fig. S61. The change in height of an initially open dandidrone that is steered to move horizontally by selective illumination of one of its sides. The change in the Z coordinate of the dandidrone upon the different light intensities. Wind tunnel speed: 0.6 m s^{-1}.

Model	Actuator	Blocking force (mN)	Power density (W kg⁻¹)	Response time (s)	Weight (mg)	Length (mm)
Active fliers	Piezoelectric bimorph (*47*)	135	165	1/110	40	143
Active fliers	Dielectric elastomer (*3*)	200	600	1/280	155	8
Active fliers	Electromagnetic actuator (*4*)	200	-	0.025	414	39
Active fliers	Graphene/Agar/Silk fibroin composite material (*6*)	-	-	0.650	5.3	10
Passive fliers	Polyimide/ low-density polyethylene bimorph (*26*)	-	-	1~2.5	4	12
Passive fliers	LCE bending strip, this study	0.5~3	0.11	~1	1.2	4

Table S1. Comparison of different actuation mechanisms.

Captions for Movies

Movie S1.

Light-driven vertical motion of a dandidrone. This real-time video demonstrates the vertical ascent or descent of both dandidrone configurations under uniform illumination. Light intensity is 700 mW cm^{-2}.

Movie S2.

Light-driven lateral motion of a dandidrone. This real-time video captures the horizontal movement of both dandidrone configurations under asymmetric illumination. Light intensity is 700 mW cm^{-2}.

Movie S3.

CFD simulation of a dandidrone with symmetric structure. The isosurfaces of Q-criterion (Qd^2/u_t^2) coloured by vorticity ($\omega_x d/t$), ranging from −5 (blue) to +5 (red), from high-fidelity CFD simulations exhibiting (top left) an unsteady periodic wake at $Da= 10^{-1}$, (top right) a steady wake at $Da=10^{-3}$, (bottom) a highly unsteady wake with vortex shedding at $Da=10^{-6}$.

Movie S4.

Vorticity contours of a dandidrone. Vorticity contours obtained by PIV at 0.1 × real-time speed (images are acquired at 500 fps and the video is played at 50 fps): (**A**) fully open (180° opening angle) dandidrone; (**B**) asymmetrically oriented dandidrone with 120° opening angle; (**C**) fully open dandidrone with 1 cm disk in the centre; (**D**) 1 cm disk.

Movie S5.

Light-induced flipping motion of a dandidrone in midair. This real-time video shows the controlled flipping motion of the dandidrone upon light stimulation. Light intensity is 700 mW cm^{-2}.

Movie S6.

Light-driven 2D manoeuvring of a dandidrone. In this video, directed light stimuli are used to manoeuvre dandidrone samples (1 and 4) within a plane, forming the letter "A" in coordination with reference samples (2, 3, and 5). The video is played at 5× speed. Light intensity is 700 mW cm^{-2}.

Movie S7.

Light controlled swarms of dandidrones. This video shows that two or three dandidrones can be guided to converge, establishing a stable triadic formation that functions as a single, coordinated group for moving. The video is played at 5× speed. Light intensity is 700 mW cm^{-2}.

Movie S8.

Light induced 3D manoeuvring of dandidrones. This video shows the dandidrone's controlled ascent to a predefined altitude, sustained horizontal navigation, and precise descent back to its original position under light stimulation. The video is played at 3× speed. Light intensity is 700 mW cm^{-2}.

Movie S9.

CFD simulation of a dandidrone with asymmetric structure. The isosurfaces of Q-criterion (Qd^2/u_t^2) coloured by vorticity ($\omega_x d/t$), ranging from −5 (blue) to +5 (red), from high-fidelity CFD simulations exhibiting (top left) a steady wake for symmetric dandidrone with opening angle of 180°, (top right) a steady wake for asymmetric dandidrone with opening angle of 150°, (bottom) a steady wake for asymmetric dandidrone with opening angle of 120°.

REFERENCES

1. M. Karásek, F. T. Muijres, C. De Wagter, B. D. W. Remes, G. C. H. E. de Croon, A tailless aerial robotic flapper reveals that flies use torque coupling in rapid banked turns. *Science* **361**, 1089–1094 (2018).

2. R. J. Wood, The first takeoff of a biologically inspired at-scale robotic insect. *IEEE Trans. Robot.* **24**, 341–347 (2008).

3. Y. Chen, H. Zhao, J. Mao, P. Chirarattananon, E. F. Helbling, N. S. P. Hyun, D. R. Clarke, R. J. Wood, Controlled flight of a microrobot powered by soft artificial muscles. *Nature* **575**, 324–329 (2019).

4. W. Shen, J. Peng, R. Ma, J. Wu, J. Li, Z. Liu, J. Leng, X. Yan, M. Qi, Sunlight-powered sustained flight of an ultralight micro aerial vehicle. *Nature* **631**, 537–543 (2024).

5. N. T. Jafferis, E. F. Helbling, M. Karpelson, R. J. Wood, Untethered flight of an insect-sized flapping-wing microscale aerial vehicle. *Nature* **570**, 491–495 (2019).

6. D. Wang, Z. Chen, M. Li, Z. Hou, C. Zhan, Q. Zheng, D. Wang, X. Wang, M. Cheng, W. Cheng, W. Hu, B. Dong, F. Shi, M. Sitti, Bioinspired rotary flight of light-driven composite films. *Nat. Commun.* **14**, 5070 (2023).

7. K. Y. Ma, P. Chirarattananon, S. B. Fuller, R. J. Wood, Controlled flight of a biologically inspired, insect-scale robot. *Science* **340**, 603–607 (2013).

8. H. V. Phan, H. C. Park, D. Floreano, Passive wing deployment and retraction in beetles and flapping microrobots. *Nature* **632**, 1067–1072 (2024).

9. A. Ramezani, S.-J. Chung, S. Hutchinson, A biomimetic robotic platform to study flight specializations of bats. *Sci. Robot.* **2**, eaal2505 (2017).

10. Y. Zhao, Z. Liu, P. Shi, C. Chen, Y. Alsaid, Y. Yan, X. He, Antagonistic-contracting high-power photo-oscillators for multifunctional actuations. *Nat. Mater.* **24**, 116–124 (2025).

11. S. Kim, Y. H. Hsiao, Y. Lee, W. Zhu, Z. Ren, F. Niroui, Y. Chen, Laser-assisted failure recovery for dielectric elastomer actuators in aerial robots. *Sci. Robot.* **8**, eadf4278 (2023).

12. Y. M. Chukewad, J. James, A. Singh, S. Fuller, RoboFly: An insect-sized robot with simplified fabrication that is capable of flight, ground, and water surface locomotion. *IEEE Trans. Robot.* **37**, 2025–2040 (2021).

13. Z. Huang, S. Li, J. Jiang, Y. Wu, L. Yang, Y. Zhang, Biomimetic flip-and-flap strategy of flying objects for perching on inclined surfaces. *IEEE Robot. Autom. Lett.* **6**, 5199–5206 (2021).

14. D. Floreano, R. J. Wood, Science, technology and the future of small autonomous drones. *Nature* **521**, 460–466 (2015).

15. H. V. Phan, H. C. Park, Insect-inspired, tailless, hover-capable flapping-wing robots: Recent progress, challenges, and future directions. *Prog. Aerosp. Sci.* **111**, 100573 (2019).

16. M. Seale, N. Nakayama, From passive to informed: Mechanical mechanisms of seed dispersal. *New Phytol.* **225**, 653–658 (2020).

17. R. Nathan, G. G. Katul, H. S. Horn, S. M. Thomas, R. Oren, R. Avissar, S. W. Pacala, S. A. Levin, Mechanisms of long-distance dispersal of seeds by wind. *Nature* **418**, 409–413 (2002).

18. S. Bai, Q. He, P. Chirarattananon, A bioinspired revolving-wing drone with passive attitude stability and efficient hovering flight. *Sci. Robot.* **7**, eabg5913 (2022).

19. D. Lentink, W. B. Dickson, J. L. van Leeuwen, M. H. Dickinson, Leading-edge vortices elevate lift of autorotating plant seeds. *Science* **324**, 1438–1440 (2009).

20. C. Cummins, M. Seale, A. Macente, D. Certini, E. Mastropaolo, I. M. Viola, N. Nakayama, A separated vortex ring underlies the flight of the dandelion. *Nature* **562**, 414–418 (2018).

21. I. M. Viola, N. Nakayama, Flying seeds. *Curr. Biol.* **32**, R204–R205 (2022).

22. B. H. Sun, X.-L. Guo, Drag scaling law and parachute terminal velocity of the dandelion. *AIP Adv.* **13**, 085305 (2023).

23. V. Iyer, H. Gaensbauer, T. L. Daniel, S. Gollakota, Wind dispersal of battery-free wireless devices. *Nature* **603**, 427–433 (2022).

24. B. H. Kim, K. Li, J. T. Kim, Y. Park, H. Jang, X. Wang, Z. Xie, S. M. Won, H. J. Yoon, G. Lee, W. J. Jang, K. H. Lee, T. S. Chung, Y. H. Jung, S. Y. Heo, Y. Lee, J. Kim, T. Cai, Y. Kim, P. Prasopsukh, Y. Yu, X. Yu, R. Avila, H. Luan, H. Song, F. Zhu, Y. Zhao, L. Chen, S. H. Han, J. Kim, S. J. Oh, C. H. Lee, Y. Huang, L. P. Chamorro, Y. Zhang, J. A. Rogers, Three-dimensional electronic microfliers inspired by wind-dispersed seeds. *Nature* **597**, 503–510 (2021).

25. J. Yang, M. R. Shankar, H. Zeng, Photochemically responsive polymer films enable tunable gliding flights. *Nat. Commun.* **15**, 4684 (2024).

26. Y. Chen, C. Valenzuela, X. Zhang, X. Yang, L. Wang, W. Feng, Light-driven dandelion-inspired microfliers. *Nat. Commun.* **14**, 3036 (2023).

27. J. T. Kim, H. J. Yoon, S. Cheng, F. Liu, S. Kang, S. Paudel, D. Cho, H. Luan, M. Lee, G. Jeong, J. Park, Y. T. Huang, S. E. Lee, M. Cho, G. Lee, M. Han, B. H. Kim, J. Yan, Y. Park, S. Jung, L. P. Chamorro, J. A. Rogers, Functional bio-inspired hybrid fliers with separated ring and leading edge vortices. *PNAS Nexus* **3**, pgae110 (2024).

28. S. Mariani, K. Cikalleshi, M. Ronzan, C. Filippeschi, G. A. Naselli, B. Mazzolai, A biodegradable, porous flier inspired by a parachute-like *Tragopogon* fruit for environmental preservation. *Small* **21**, e2403582 (2025).

29. K. Johnson, V. Arroyos, A. Ferran, R. Villanueva, D. Yin, T. Elberier, A. Aliseda, S. Fuller, V. Iyer, S. Gollakota, Solar-powered shape-changing origami microfliers. *Sci. Robot.* **8**, eadg4276 (2023).

30. J. Yang, H. Zhang, A. Berdin, W. Hu, H. Zeng, Dandelion-inspired, wind-dispersed polymer-assembly controlled by light. *Adv. Sci.* **10**, e2206752 (2023).

31. T. H. Ware, M. E. McConney, J. J. Wie, V. P. Tondiglia, T. J. White, Voxelated liquid crystal elastomers. *Science* **347**, 982–984 (2015).

32. T. J. White, D. J. Broer, Programmable and adaptive mechanics with liquid crystal polymer networks and elastomers. *Nat. Mater.* **14**, 1087–1098 (2015).

33. H. Zeng, O. M. Wani, P. Wasylczyk, R. Kaczmarek, A. Priimagi, Self-regulating iris based on light-actuated liquid crystal elastomer. *Adv. Mater.* **29**, 1701814 (2017).

34. S. Li, D. Pan, L. Zeng, J. Li, X. Shao, Flow over a radiating multi-filamentous structure with various opening angles: From disk-like to cone-like shape. *Phys. Fluids* **36**, 033622 (2024).

35. M. Seale, O. Zhdanov, M. B. Soons, C. Cummins, E. Kroll, M. R. Blatt, H. Z. Behtash, A. Busse, E. Mastropaolo, J. M. Bullock, I. M. Viola, Environmental morphing enables informed dispersal of the dandelion diaspore. *eLife* **11**, e81962 (2022).

36. S. Li, D. Pan, J. Li, X. Shao, Drag and wake structure of a quasi-dandelion pappus model at low and moderate Reynolds numbers: The effects of filament width. *Phys. Fluids* **33**, 121904 (2021).

37. A. R. Shenoy, C. Kleinstreuer, Flow over a thin circular disk at low to moderate Reynolds numbers. *J. Fluid Mech.* **605**, 253–262 (2008).

38. X. Tian, M. C. Ong, J. Yang, D. Myrhaug, Large-eddy simulations of flow normal to a circular disk at $Re = 1.5 \times 10^5$. *Comput. Fluids* **140**, 422–434 (2016).

39. M. Barbara, E. D. Dottore, T. Speck, N. Rowe, C. Laschi, Energy-saving movement strategies in animals and plants for robot design. *Nat. Rev. Bioeng.* **3**, 921–938 (2025).

40. L. Chang, D. Wang, Z. Huang, C. Wang, J. Torop, B. Li, Y. Wang, Y. Hu, A. Aabloo, A versatile ionomer-based soft actuator with multi-stimulus responses, self-sustainable locomotion, and photoelectric conversion. *Adv. Funct. Mater.* **33**, 2212341 (2023).

41. G. E. Bauman, J. M. McCracken, T. J. White, Actuation of liquid crystalline elastomers at or below ambient temperature. *Angew. Chem. Int. Ed. Engl.* **61**, e202202577 (2022).

42. Global Climate Observing System (GCOS), "The Status of the Global Climate Observing System 2021: The GCOS Status Report" (GCOS-240, World Meteorological Organization, 2021).

43. K. E. Madsen, M. T. Flavin, J. A Rogers, Materials advances for distributed environmental sensor networks at scale. *Nat. Rev. Mater.* **11**, 26–49 (2026).

44. M. Barbara, S. Mariani, M. Ronzan, L. Cecchini, I. Fiorello, K. Cikalleshi, L. Margheri, Morphological computation in plant seeds for a new generation of self-burial and flying soft robots. *Front. Robot. AI* **8**, 797556 (2021).

45. N. He, K. W. Kwan, A. H. W. Ngan, Enhancing the light-induced actuation of nickel oxyhydroxide by aluminum doping. *Adv. Mater. Technol.* **10**, 2401263 (2025).

46. I. M. Viola, A. Potnis, S. Bhattacharyya, E. J. Williams, D. Halley, D. Murphy, An accelerating wind tunnel for testing untethered bodies in transverse gusts. *Exp. Fluids* **66**, 205 (2025).

47. R. J. Wood, "Design, fabrication, and analysis of a 3DOF, 3cm flapping-wing MAV," in *2007 IEEE/RSJ International Conference on Intelligent Robots and Systems* (IEEE, 2007), pp. 1576–1581.
